# Author Correction: How machine learning can help select capping layers to suppress perovskite degradation

**DOI:** 10.1038/s41467-020-19655-3

**Published:** 2020-11-03

**Authors:** Noor Titan Putri Hartono, Janak Thapa, Armi Tiihonen, Felipe Oviedo, Clio Batali, Jason J. Yoo, Zhe Liu, Ruipeng Li, David Fuertes Marrón, Moungi G. Bawendi, Tonio Buonassisi, Shijing Sun

**Affiliations:** 1 Massachusetts Institute of Technology, 77 Massachusetts Avenue, Cambridge, MA 02139 USA; 2 National Synchrotron Light Source II, Brookhaven National Laboratory, Upton, NY 11973 USA; 3 Instituto de Energía Solar-ETSIT, Universidad Politécnica de Madrid, 28040 Madrid, Spain

**Keywords:** Materials chemistry, Materials for energy and catalysis, Solar cells

Correction to: *Nature Communications* 10.1038/s41467-020-17945-4, published online 20 August 2020.

The original version of this Article contained an error in Fig. 4a, in which the value for the ‘partition coefficient’ for phenyltriethylammonium was incorrectly given as 0. The correct value for the ‘partition coefficient’ is 2.9.

This has been corrected in both the PDF and HTML versions of the Article.

