# Supplementary material for: How machine learning can help select capping layers to suppress perovskite degradation
Source: Nat Commun. 2020 Aug 20;11:4172. doi: 10.1038/s41467-020-17945-4 (PMC7441172; doi:10.1038/s41467-020-17945-4)
Supplement: Supplementary file 1 — Supplementary Information [file 41467_2020_17945_MOESM1_ESM.pdf]

Supplementary Information

How machine learning can help select capping layers to suppress perovskite degradation

Hartono et al.

Supplementary Methods

The degradation onset extraction

The degradation onset is extracted the following way.

a. We started by performing accelerated degradation test until all the materials decompose, instead of set a fixed period of time and examine whether a decomposition has taken place. Hence, all the materials would have been classified as 'yes' in a conventional binary classification model.
b. We built a model to predict the degradation onset, i.e. the time when the decomposition starts. This is a prediction on how long will a film remain structurally stable (in black perovskite phase) under 85% relative humidity/ 85°C test condition. Considering that the color change corresponds to the change of crystal structure from $MAPbI_3$ into PbI_2,[2,3] as soon as the yellow spots appear, the films have started to degrade. The variable, time, is a continuous variable.
c. We can also predict kinetics of the decomposition (time constant) by extracting the rate of change in color after the degradation starts (red slope in Supplementary Figure 3), however, in this study, we focused on only taking the time at the onset when the degradation starts as our output.

Machine learning framework and regression models

All the machine learning models used are using the scikit-learn library[1] in Python. 6 machine learning models are trained, including linear regression (LR), K-nearest neighbor regression (KNN), random forest regression (RF), gradient boosting regression with decision trees (GB), neural network (multilayer perceptron) regression (NN), and support vector machine regression (SVR). The LR is ordinary least squares linear regression, which minimizes the residual sum of squares between the experimental/ observed data points and the predicted data points. This serves as the benchmark of the other 5 algorithms. The other 5 algorithms have their parameters optimized based on their accuracy, using cross-validated root mean square error (RMSE), with 80% : 20% training : test split. The cross-validation helps to see how the results of trained models will generalize to an independent dataset.

We performed normalization of the model inputs (X) using the the StandardScaler algorithm, in the scikit-learn library, which calibrates the mean and scales to unit variance. The inputs of the tree-based algorithms, however, do not need normalization. Therefore, we consider both the normalized/ non-normalized input (X), and compared their cross-validated RMSE.

The parameters for the machine learning algorithms and their RMSE's are provided below (Supplementary Table 2 and 3). The linear regression has a significant difference between of cross-validated RMSE between the normalized and non-normalized data (Supplementary Figure 5), while the other algorithms are not affected much by the data pre-processing method.

The regression models work by fitting the input (X) consisting of molecular properties of the capping layer materials and their processing conditions (the precursor solution concentration and

the annealing temperature), into the output (y), consisting of the degradation parameters (in this study, the time-intercept of red color value/ R).

Since the shape of the time-dependent RGB curves extracted from the images are relatively the same across different capping layers (Supplementary Figure 3, Supplementary Figure 37), the different options to characterize the degradation are the time-intercept/ the onset of color change, and the speed of color change. Considering that the color change corresponds to the change of crystal structure from $MAPbI_3$ into PbI_2,[2,3] as soon as the yellow spots appear, the films have started to degrade. Therefore, we choose the time-intercept of the degradation curve as the sign of degradation starting point in the film.

Supplementary Figure 35 shows that there is a weak inverse correlation between the red (R) slope with the red (R) time-intercept (Pearson correlation value = -0.61). The more stable capping materials with high time-intercepts (above 260 minutes) show a stronger inverse correlation than the less stable materials (time-intercepts below 250 minutes). For more stable materials, we can also conclude that they tend to have lower slope/ slower 'yellowing' speed.

Supplementary Figure 37 shows that the change in blue color is insignificant in comparison to the red and green color change. This is supported by the fact that the $MAPbI_3$ films undergo yellowing when they turn into PbI_2. Yellow comes from red and green light. Therefore, we can choose between red and green to describe the degradation of the films. Supplementary Figure 4 shows how the red and green color correlates for both the time-intercept and the slope of degradation curves. Pearson correlation values for time-intercept and slope are 0.99 and 0.96, respectively, indicating that we can choose either color as our dataset's degradation descriptor. Hence, in this study, we choose to describe the degradation process using the time-intercept of the red color curve.

Feature engineering

We only include 14 descriptors/ features/ molecular properties from the PubChem 2019 database. There are more molecular features from the database that is not included, because the molecules that we explore have the same values in those features, such as the number of Hydrogen bond acceptor; the number of other atoms (O, Cl, F); formal charge; atom, bond stereocenter count; and covalently-bonded unit count.

The molecular features' correlations between each other is shown in Supplementary Figure 10, using Pearson correlation value, which ranges between -1.00 and 1.00. There are few things that should be noted:

1. The Pearson correlation value between the number of Br and I atoms is -1.00, and this is due to the fact that the X-site anion is either Br or I.
2. The molecular weight is correlated strongly (> 0.9) with the number of heavy atoms, and the number of C atoms. The number of C atoms also correlates with the complexity, partition coefficient ($x \log P$), the number of rotatable bonds, and the number of Hydrogen atoms. These molecular properties are all related to each other.
3. The topological polar surface area and the number of hydrogen bond donor, which come as the most important feature, correlate with Pearson correlation value of 0.81.

Quantitative color analysis

The degradation of perovskite thin-films was tracked over time by photographing, that is capable of detecting degradation mechanisms changing the color of the film, in this case $MAPbI_3$ decomposition to MAI and PbI_2. A good approximation of the color of the samples is obtained with an RGB camera. In order to retrieve reproducible and repeatable quantitative color data, the setup was designed to keep the illumination conditions as stable as possible over time and the pictures were color calibrated.

ThorLabs DCC1645C camera (with removed IR filter to collect signal from a wider spectrum) with ThorLabs MVL6WA lens was used for taking in situ RGB pictures of the samples every 3 minutes during the aging test. Camera settings were kept fixed in this study and they were chosen to give as light pictures as possible without oversaturating the lightest color patch in our reference color chart, which would distort the color calibration procedure. All the samples were captured simultaneously in each picture, therefore we did not have to move the sample holder. It was painted with medium gray color to prevent disturbance from over- or undersaturation of the background of the samples, as well as designed by shape to minimize reflections from the samples to the camera. The degradation chamber was covered by light-blocking curtains to remove stray light and illuminated using Advanced Illumination LED lamp and controller. We kept a mini-scale reference color chart in the picture area during the aging tests, and by checking its color over time we were able to confirm illumination remained constant during the aging test.

The mean color calibrated color of each sample was determined from the photographs. In this work, X-Rite Color Checker Passport with 28 reference color patches was photographed at the beginning of each aging test in the aging chamber. The color data retrieved from the color patches was used for transforming the photographs of the samples to a stable reference color space (standard illuminant D50, standard observer CIE 1931 2 degrees). This way, the colors are comparable even though the pictures would be taken under different illumination conditions or with different camera-lens setups.

The approach chosen in our setup is to transform the samples into a larger L*a*b color space and to apply 3D thin-plate spline color warping that has been shown to be among the most accurate color warping methods for color calibration.[6] Distortion between the colors of the reference color chart in real and reference color spaces is defined as the following Supplementary Equation 1.

$$D = \frac{\begin{bmatrix} V \\ O(4,3) \end{bmatrix}}{\begin{bmatrix} K & P \\ P^T & O(4,4) \end{bmatrix}} \tag{1}$$

where matrix *V* represents the colors of the reference color chart in the reference space (obtained from the color chart manufacturer), matrix *P* represents them in the original space, and matrix *K* is the distortion between the colors in the reference color chart.[6] Applying the same distortion *D* to the samples completes the color warping is shown in the following Supplementary Equation 2.

$$\begin{bmatrix} V_s \\ O(4,3) \end{bmatrix} = \begin{bmatrix} K_{sr} & P_s \\ P_s^T & O(4,4) \end{bmatrix} D \tag{2}$$

where matrices V_s and P_s represent the colors of the samples in the reference and original space, respectively, and matrix K_{sr} represents the distortion between the colors of the samples and

reference color chart. After color calibration, the sample colors were transformed back to RGB space.

Finally, the "red onset" or degradation onset (Supplementary Figure 3) was calculated as time-intercept ($t_{intercept}$) of the extrapolation from red channel sharpest change. The onset can be defined as the following Supplementary Equation 3.

$$y = At + B, \text{ where } A = \left(\frac{dR}{dt}\right)_{\max} \tag{3}$$

where t is degradation time, and R is the calibrated red channel curve. Therefore, $t_{\text{intercept}} = -\frac{B}{A}$, where $y = 0$.

Supplementary Notes

Machine learning prediction and limitation

We have performed a test following the same workflow for predicting potentially high-performing capping layer material and examine its experimental performance. We completely removed the best performing capping layer material, PTEAI from the capping layer candidates, and trained the models with the remaining 20 capping layer candidates. The feature importance rank result is similar to the result shown in Figure 3d from the main text, showing that the number of hydrogen-bond donor and the topological polar surface area come out as the two most important features in random forest regression model. The model still predicts materials with low hydrogen-bond donor and polar surface area to be as the top most stable capping layer material (Supplementary Figure 16). This nevertheless points us to PTEAI as a promising candidate, since PTEAI has hydrogen bond donor of 0, and topological polar surface of 0 $Å^2$. Based on this model, the predicted degradation onset for PTEAI is 355.9 minutes, the mean experimental degradation onset for PTEAI in our study is 462 minutes, with the standard deviation of 115.

We also used the model developed in this study to predict literature reports caffeine (264.2 minutes), theobromine (121.5 minutes), and theophylline (103.2 minutes) (Supplementary Figure 36).

Based on the feature importance rank (Figure 3d from the main text) coming from the random forest regressor, to pick the organic *A*-site cation, the most important capping molecular features are the following.

1. The first thing that we should look at is how polar the area of the cation. The less polar the cation is, the more stable it is as a capping layer material for $MAPbI_3$ film. Polarity has relatively low correlation with other variables, the highest correlation is with the number of hydrogen-bond donor (Pearson correlative coefficient is 0.81).
2. Molecular weight is the next important feature, which means the cation size matter. The higher the molecular weight is, and the more complex (branches, different subgroups) the organic cation is, the more the capped-films' stability improves. The molecular weight has the highest correlations with the number of carbon atoms (Pearson correlative coefficient is 0.95), the number of heavy atoms (0.95), complexity (0.89), the number of hydrogen atoms (0.89), and partition coefficients (0.87).
3. The capping material precursor solution concentration is the third most important feature, where higher precursor solution concentration improves stability of the capped films. As the X-ray diffraction (XRD) data has shown, the capping materials form low dimensional perovskite, by reacting with the excess PbI_2 coming from the $MAPbI_3$ film.[4] Therefore, the higher the solution concentration is, the thicker the capping layer forms, and the more resistant the film is from the environmental stress.[5] However, a thicker capping layer might lead to the reduction in device performance, which warrants a future study considering the trade-off between stability and performance of capped devices.

In terms of the limitation, the fact that the perovskite degrades into yellow-phase PbI_2 is the sole reason why choosing either red or green color to characterize the film degradation works. If we are working with different types of perovskite materials (Bismuth- or Antimony-based, for

instance), the degradation product will no longer be the same, and hence, the red color usage to characterize the degradation will no longer be valid.

Another limitation comes from the fact that we are using $MAPbI_3$ as the perovskite absorber layer, which has a distinct shape, similar to a step function, as shown in Supplementary Figure 3 and 37. This allows us to characterize degradation by picking the degradation onset and slope. If we use another material as the absorber, for instance the multi-cation perovskites, it is likely that we do not get the same shape, and thus, we need to find another way to characterize how much the samples have degraded over time.

Supplementary Figures

Supplementary Figure 1. Raw, compiled results from accelerated aging tests. Comparison between average RGB values from degradation of bare $MAPbI_3$, with capping layers fabricated at optimum condition that gives maximum onsets: formamidinium iodide (FAI), guanidinium iodide (GI), ethylammonium iodide (EAI), dimethylammonium iodide (DMAI), *iso*-propylammonium iodide (iPAI), imidazolium iodide (IDI), *tert*-butylammonium iodide (tBAI), phenylammonium iodide (PhAI), phenylammonium bromide (PhABr), benzylammonium iodide (BzAI), benzylammonium bromide (BzABr), phenylethylammonium iodide (PEAI), *n*-octylammonium iodide (OAI), *n*-octylammonium bromide (OABr), phenyltriethylammonium iodide (PTEAI), dodecylammonium iodide (DAI), dodecylammonium bromide (DABr), tetrapropylammonium iodide (TPAI), tetrapropylammonium bromide (TPABr), tetrabutylammonium iodide (TBAI), and tetrabutylammonium bromide (TBABr). Depending on the processing conditions, the yellowing onset of the films happened ±30 minutes.

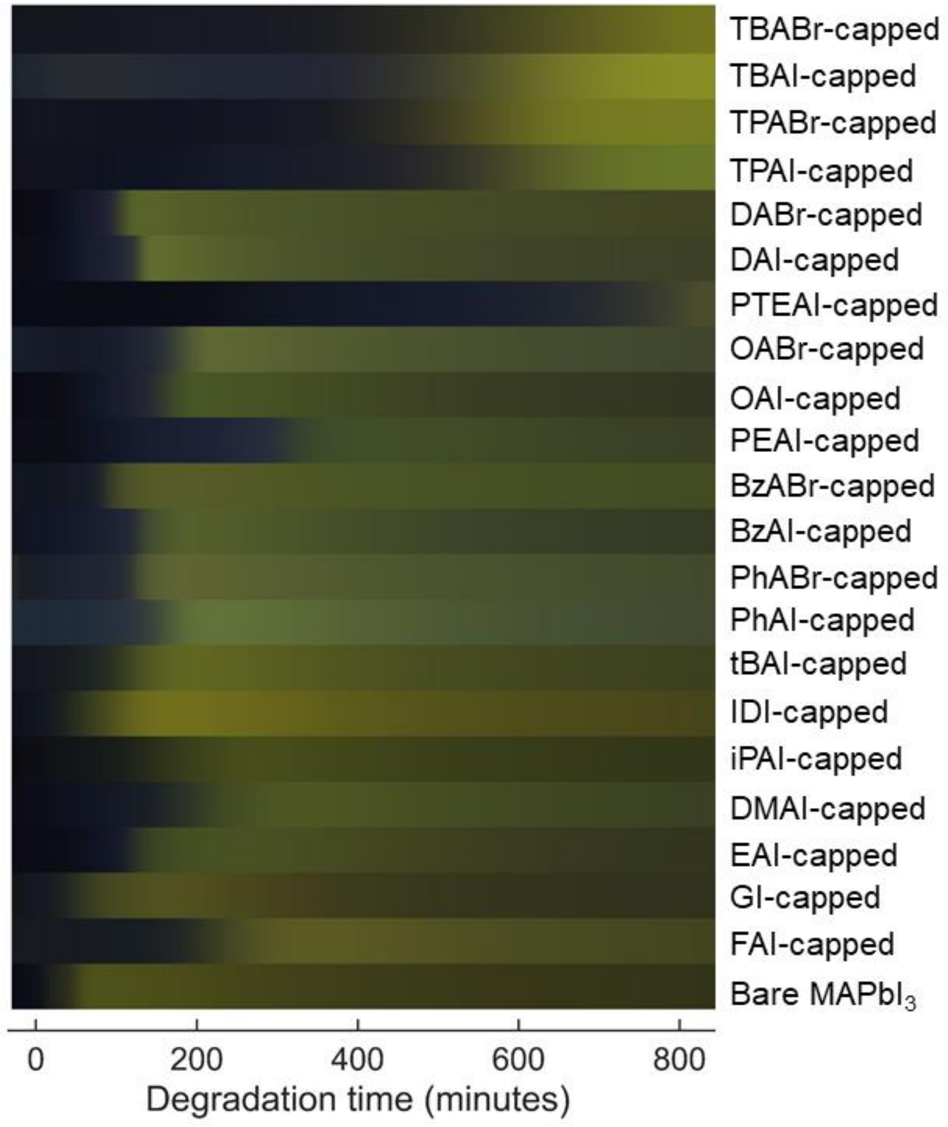

Supplementary Figure 2. The red and green channel values of optimum iodide- and bromide-based capping layers (10 mM of capping layer solution and 75°C annealing temperature for TBABr-, TBAI-, TPABr-, TPAI-, DABr-, DAI-, PTEAI-, OAI-, PEAI-, BzAI-, DMAI-, EAI-capped; 10 mM of capping layer solution and 100°C annealing temperature for OABr-, BzABr-, PhABr-, PhAI-, tBAI-, IDI-, iPAI-, GI-, FAI-capped) quantifying the data presented in Figure S1.The shaded red area shows the bare $MAPbI_3$ onset. The dotted line is the baseline ofjupy each sample.

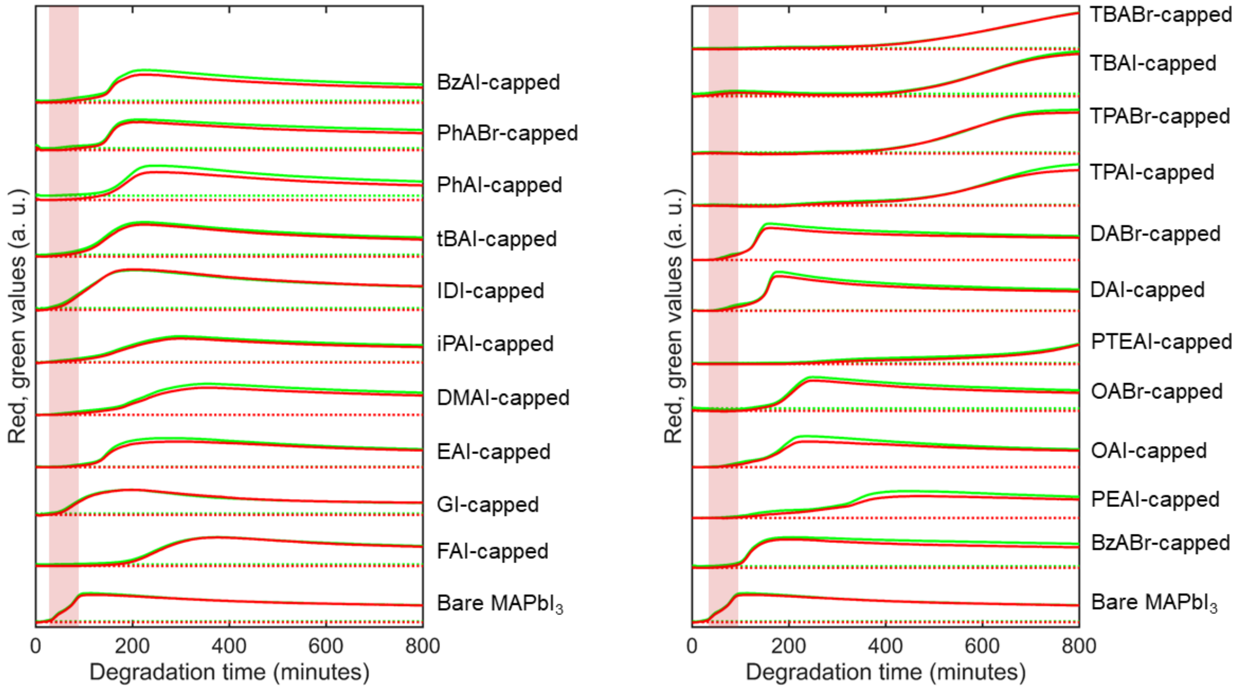

Supplementary Figure 3. The red onset (the time-intercept of the degradation curve) and the red slope (the slope of the sharpest change in the degradation curve) for the red channel.

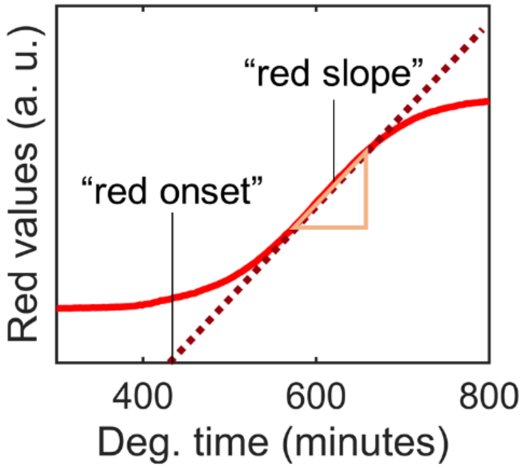

Supplementary Figure 4. The correlation between the time-intercept R and G (**a**) of all data points, with Pearson correlation value of 0.99 and between the slope of R and G (**b**) of all data points, with Pearson correlation value of 0.96.

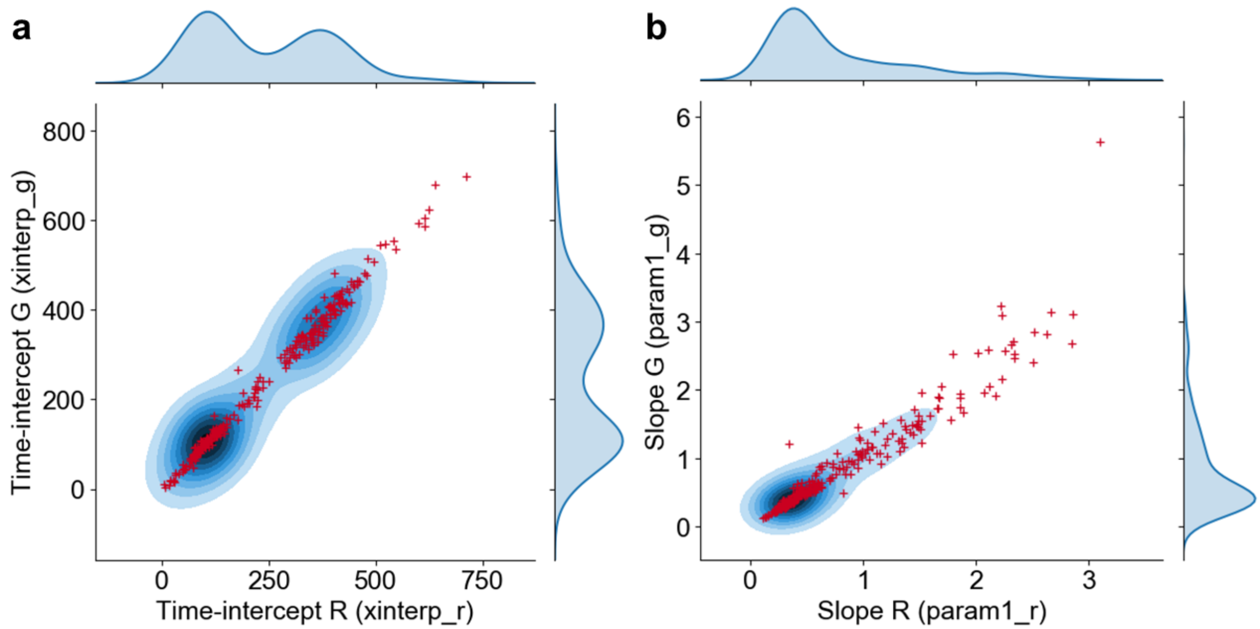

Supplementary Figure 5. Cross-validated RMSE for normalized input (**a**) and its inset (**b**), and non-normalized input (**c**). Error bars show the standard deviation of the cross-validated RMSE results.

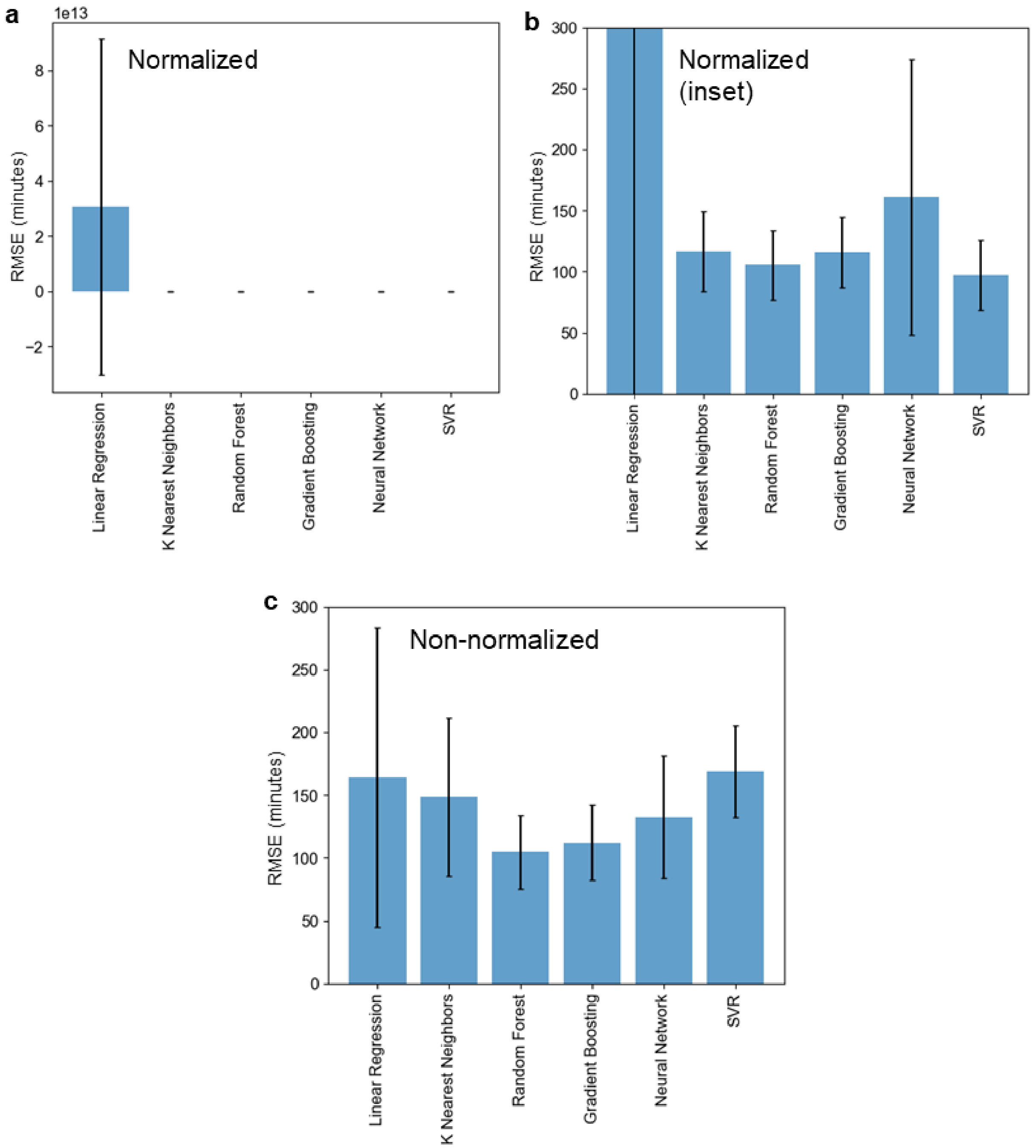

Supplementary Figure 6. The feature importance rank generated using SHAP and linear-regression (LR, non-normalized) for the molecular properties determining the time-intercept/ degradation onset of the capped films. The *x*-axis corresponds to the model output (higher means improving the stability, and vice versa). Since there are inconsistencies between the number of carbon atom and the molecular weight, we cannot use the linear regression results, even though the cross-validated RMSE is comparable to the other methods.

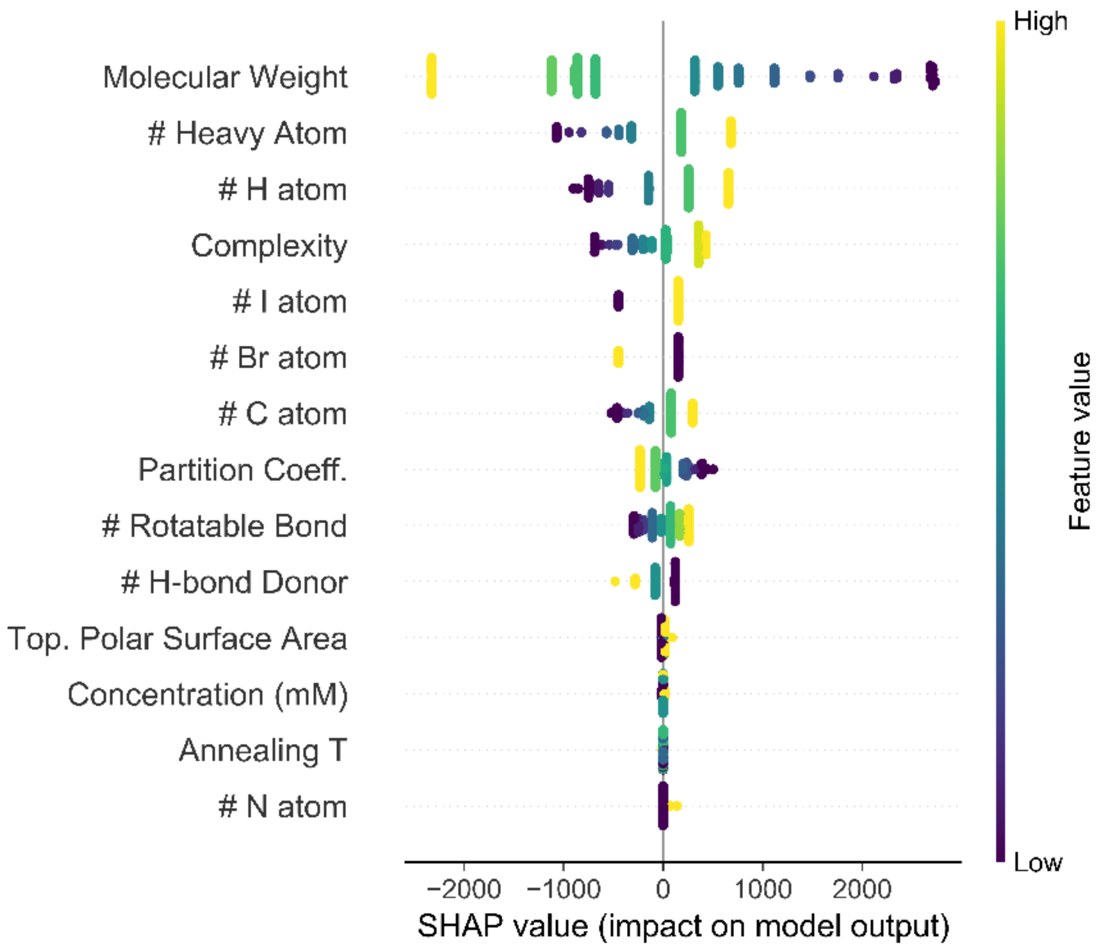

Supplementary Figure 7. The feature importance rank generated using SHAP and linear regression (LR, normalized) for the molecular properties determining the time-intercept/ degradation onset of the capped films. The *x*-axis corresponds to the model output (higher means improving the stability, and vice versa). Since there are inconsistencies between the number of carbon atom and the molecular weight, we cannot use the linear regression results, even though the cross-validated RMSE is comparable to the other methods.

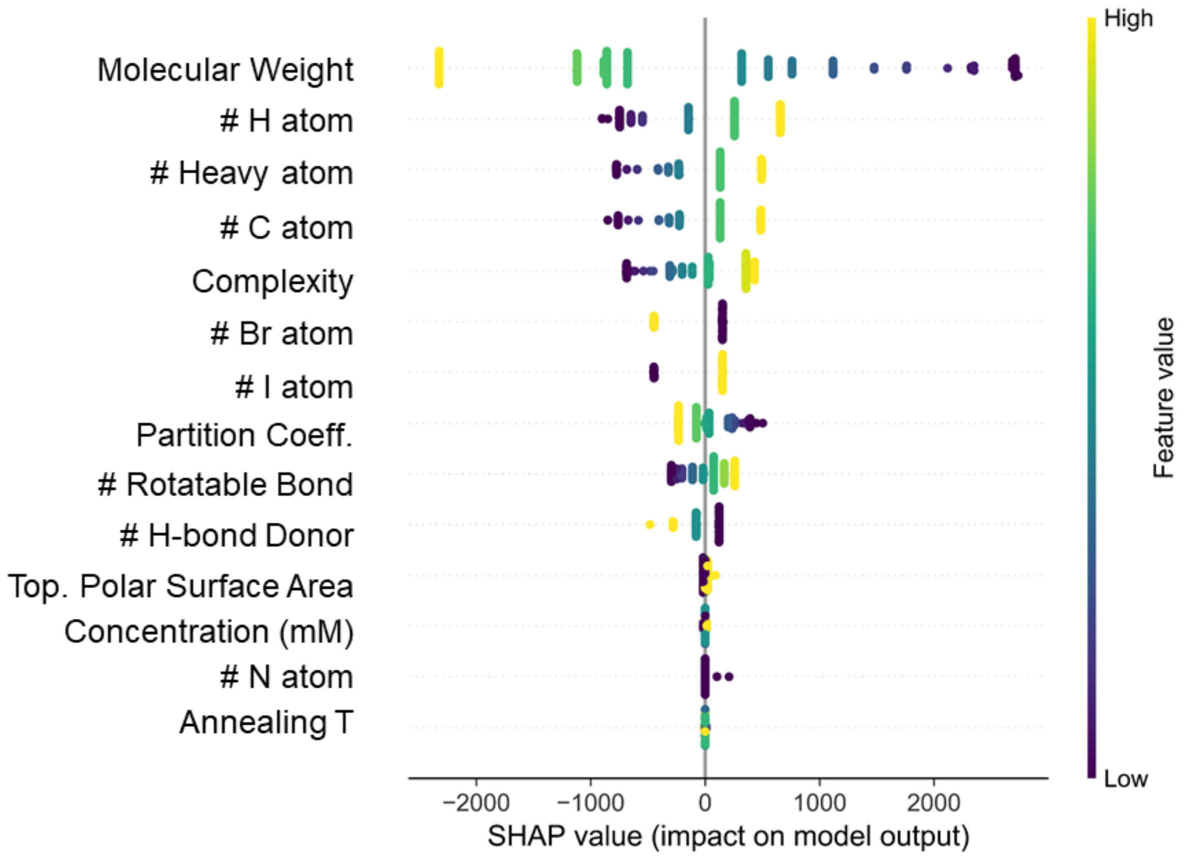

Supplementary Figure 8. The ANCOVA statistical analysis for time-intercept R data/ red onset/ *xinterp_r* at 95% confidence level. The sigma values lower than 0.05 show statistically significant values (purple color) The most stable capping layer, PTEAI, is statistically significant in comparison to other materials and bare $MAPbI_3$.

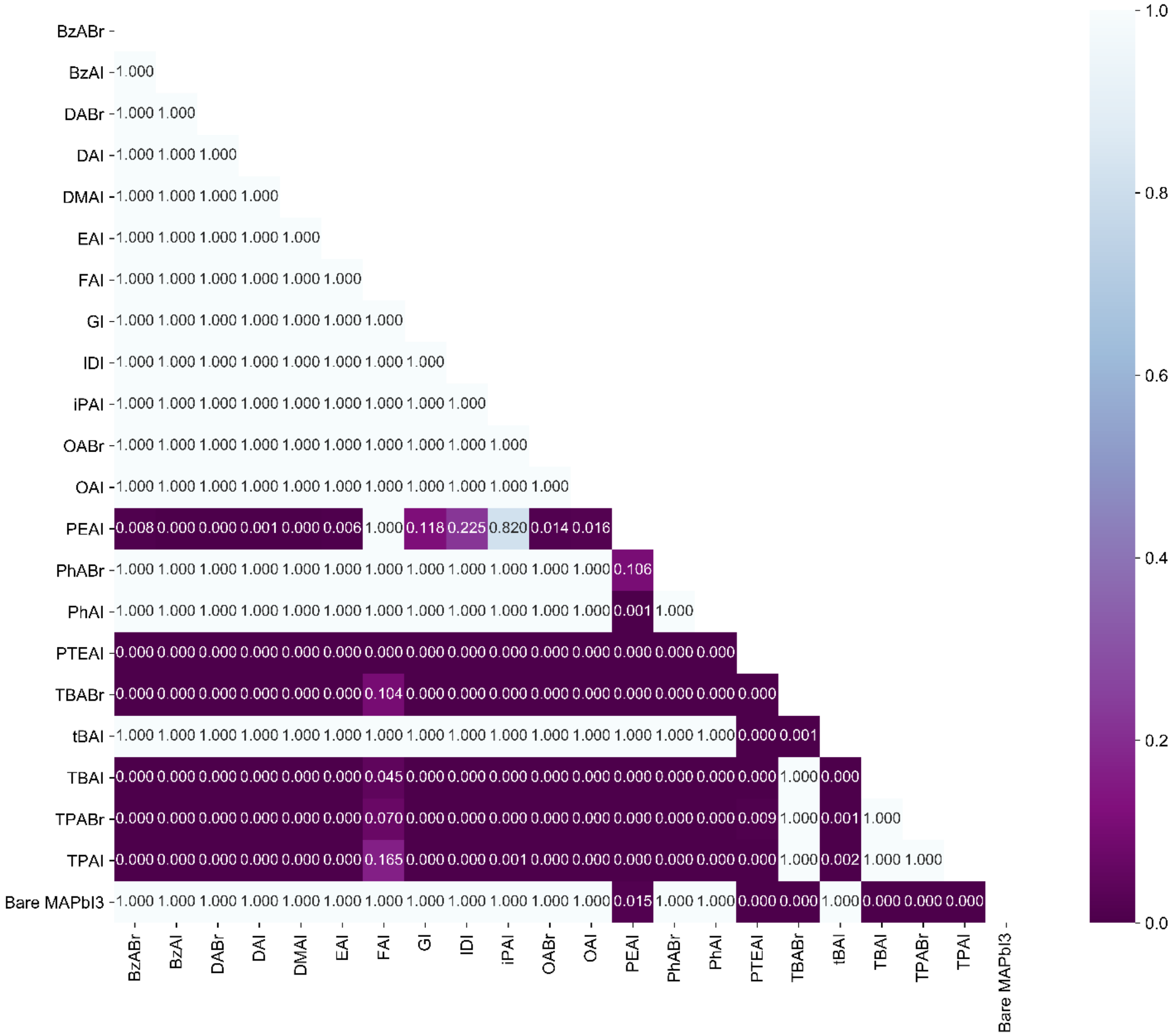

Supplementary Figure 9. The feature importance ranking obtained from the gradient boosting regression with decision trees algorithm and SHAP library, showing the chemical properties and processing conditions in descending order of importance (rank). The yellow and purple color indicate high and low values of a given feature, respectively. The top two features from this model, number of hydrogen bond donor and topological polar surface area are the same as the top two features from random forest regression model.

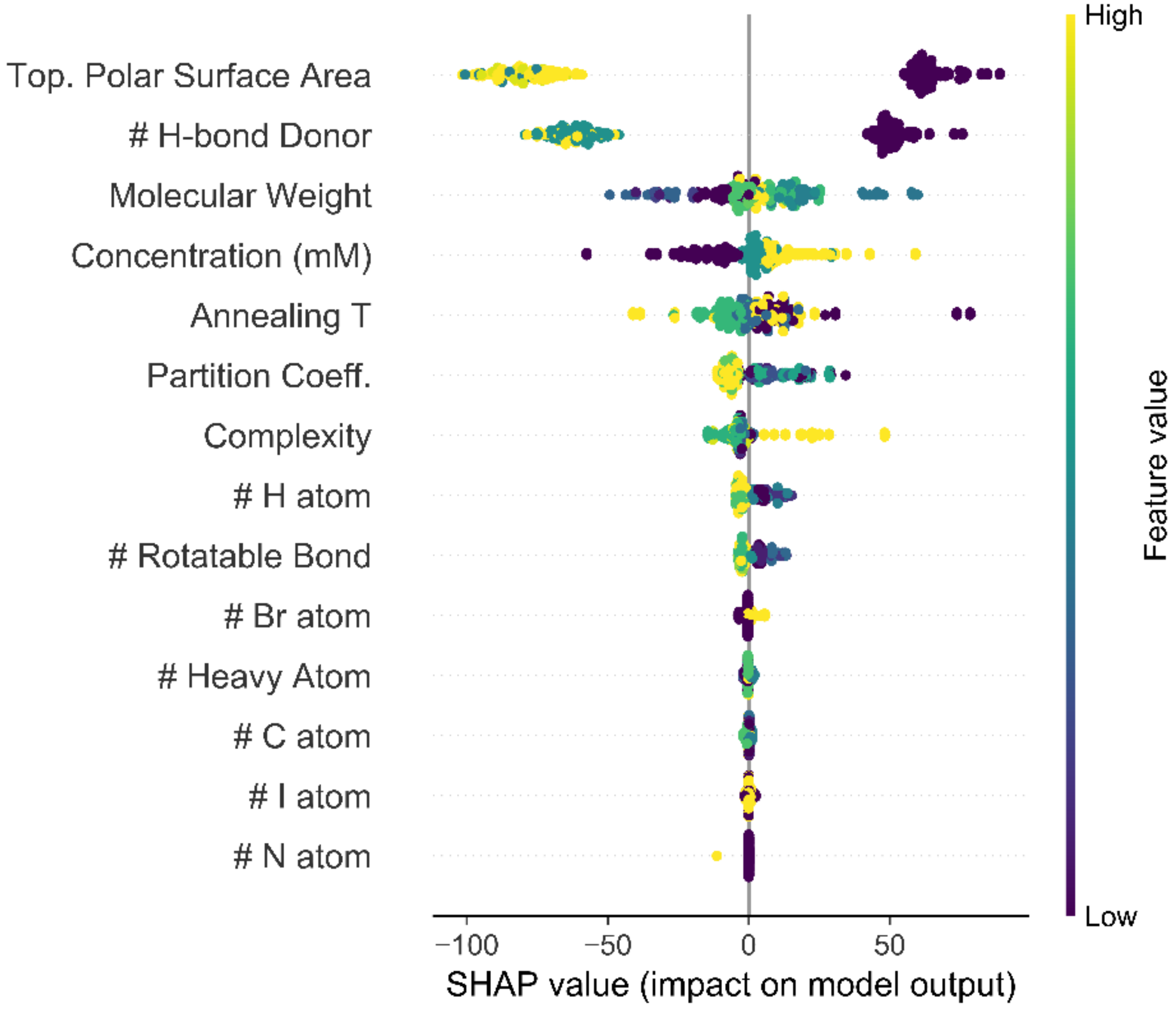

Supplementary Figure 10. The Pearson correlative coefficients for the machine learning input (*X*), which includes processing conditions and the molecular properties. The negative values imply an inverse relationship.

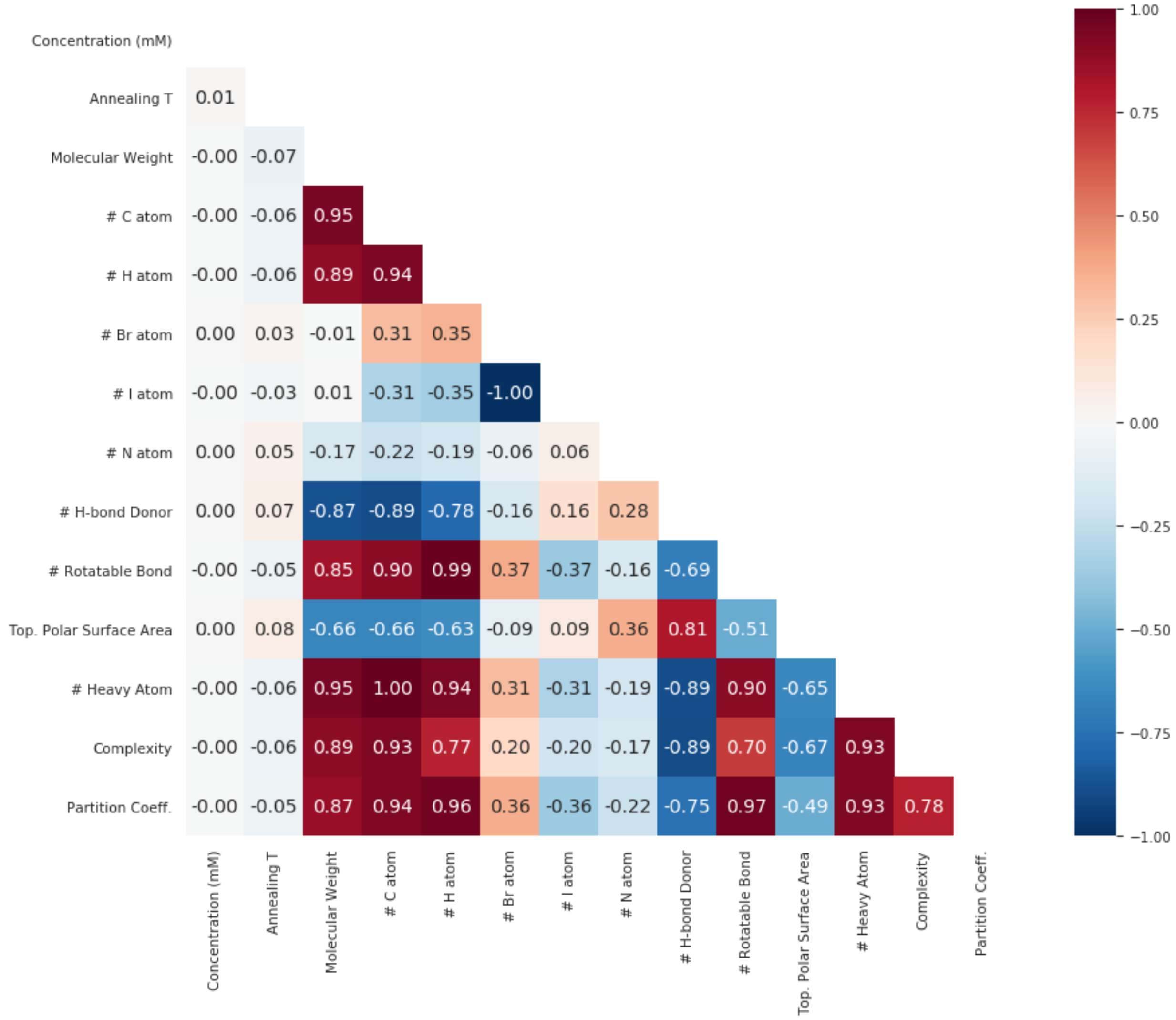

Supplementary Figure 11. N,N,N-trimethylnaphthalen-1-aminium iodide, as a possible capping layer material to explore.

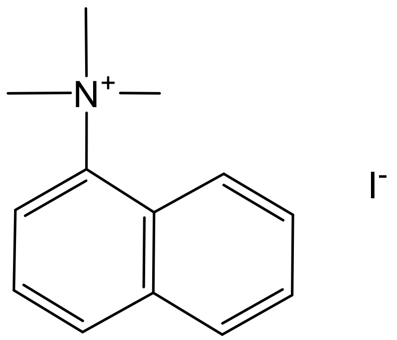

Supplementary Figure 12. The feature importance rank generated using SHAP and multi-layer perceptron neural network (NN, non-normalized) for the molecular properties determining the time-intercept/ degradation onset of the capped films. The *x*-axis corresponds to the model output (higher means improving the stability, and vice versa).

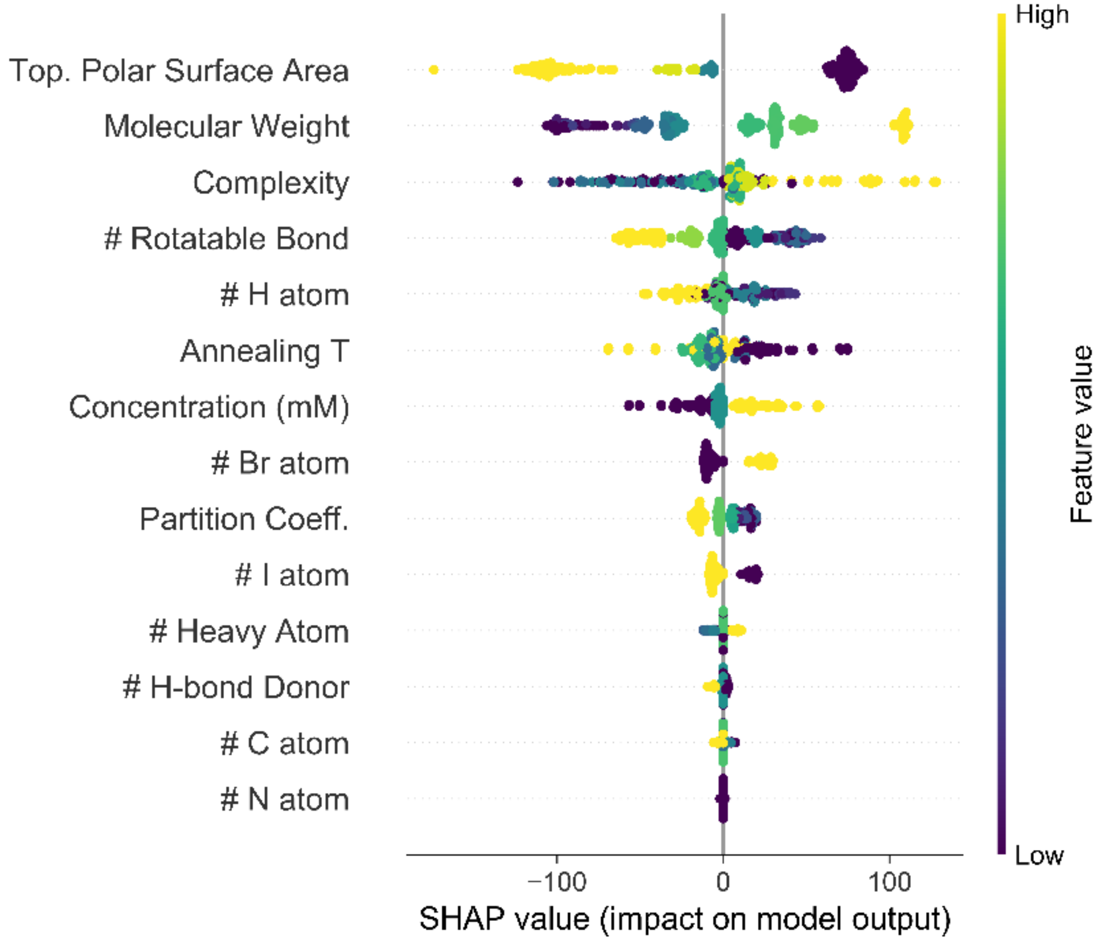

Supplementary Figure 13. The feature importance rank generated using SHAP and support vector machine regression (SVR, non-normalized) for the molecular properties determining the time-intercept/ degradation onset of the capped films. The *x*-axis corresponds to the model output (higher means improving the stability, and vice versa).

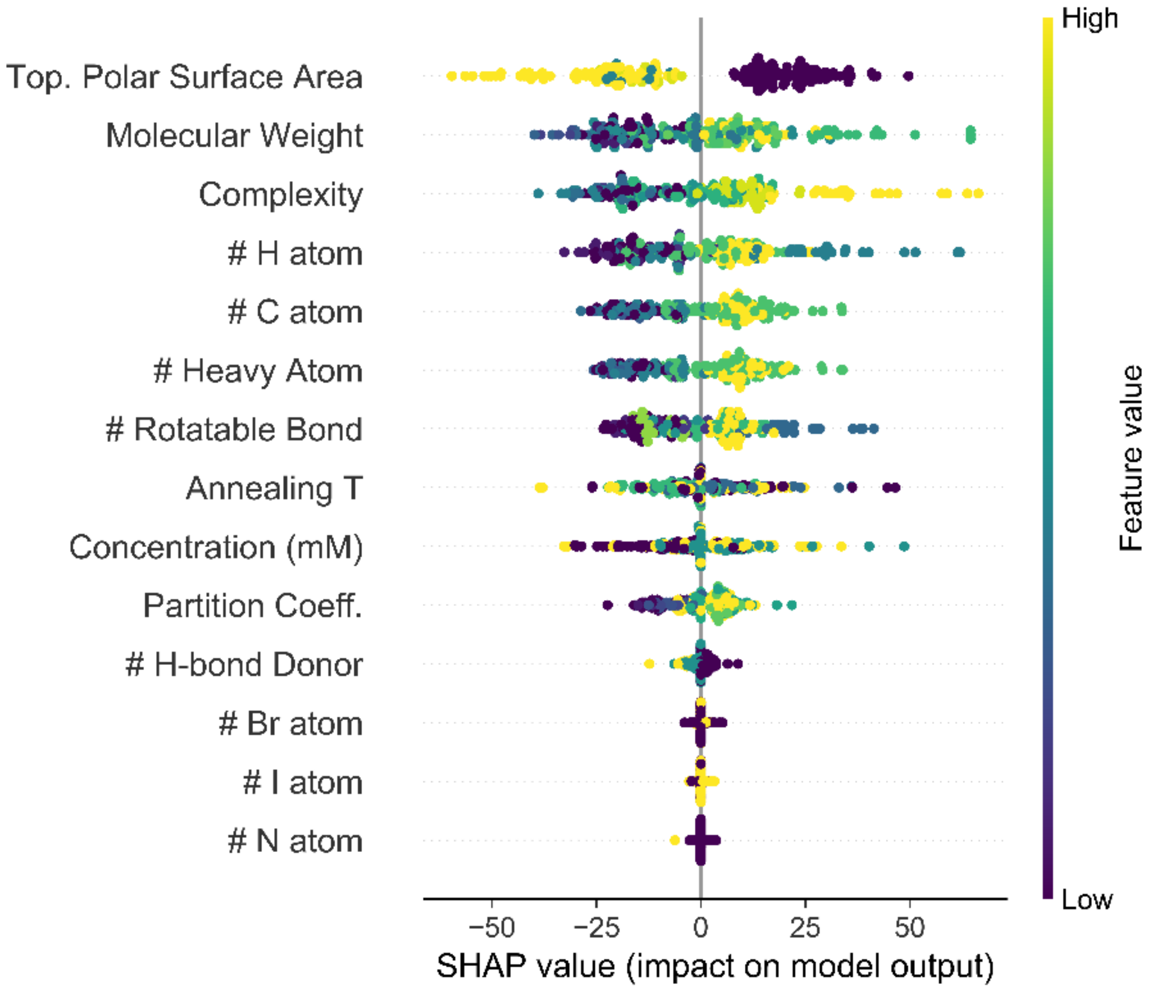

Supplementary Figure 14. The feature importance rank generated using SHAP and multi-layer perceptron neural network (NN, normalized) for the molecular properties determining the time-intercept/ degradation onset of the capped films. The *x*-axis corresponds to the model output (higher means improving the stability, and vice versa).

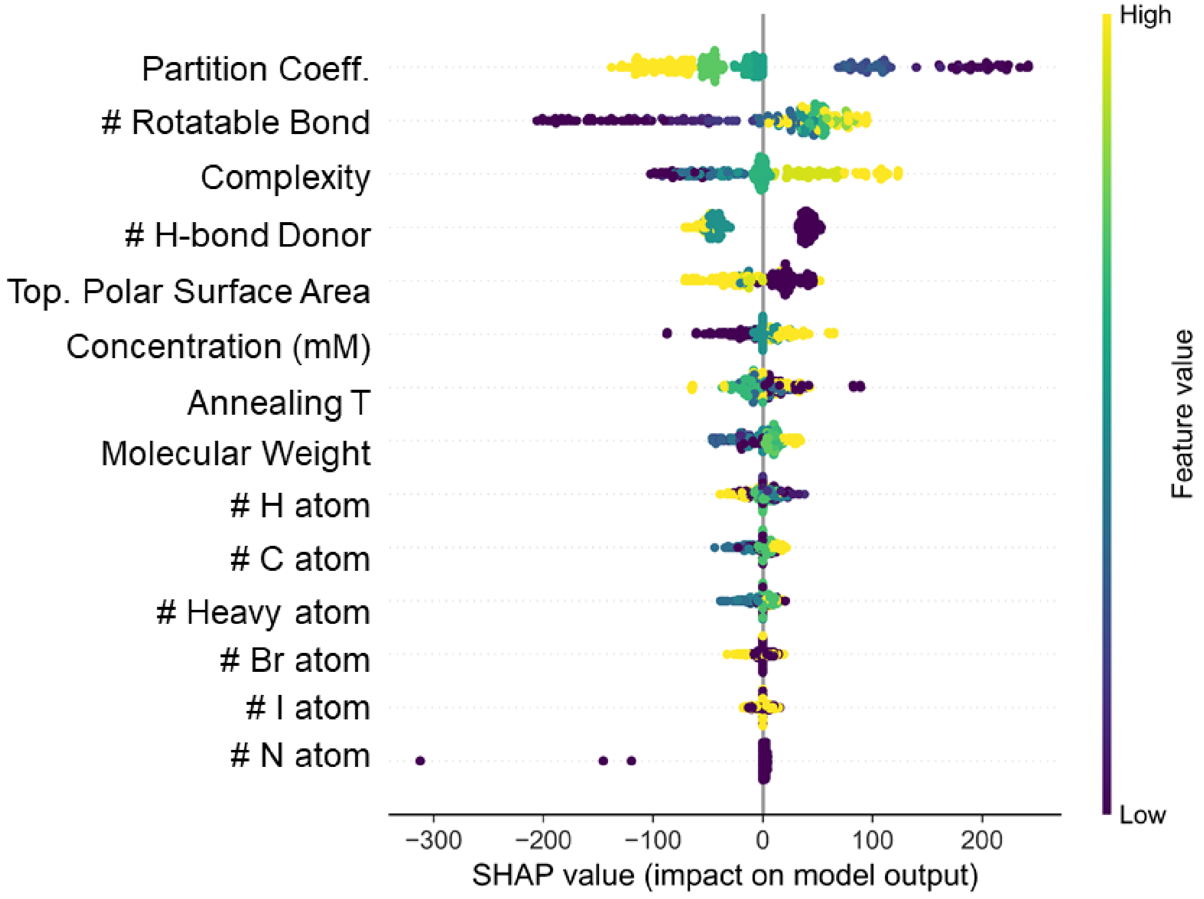

Supplementary Figure 15. The feature importance rank generated using SHAP and support vector machine regression (SVR, normalized) for the molecular properties determining the time-intercept/ degradation onset of the capped films. The *x*-axis corresponds to the model output (higher means improving the stability, and vice versa).

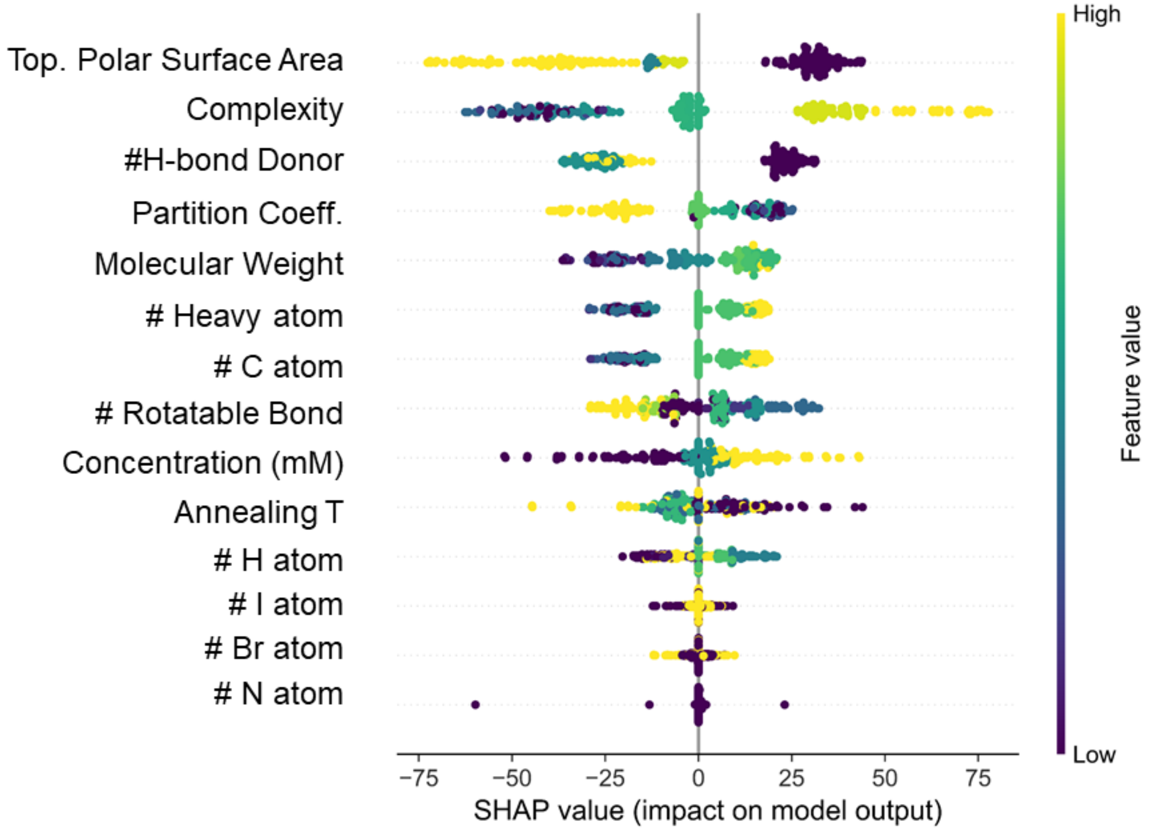

Supplementary Figure 16. The feature importance rank generated using SHAP and random forest regression (RF, non-normalized) for the molecular properties determining the time-intercept/ degradation onset of the capped films, and excluding the top stable capping layer material, PTEAI. The *x*-axis corresponds to the model output (higher means improving the stability, and vice versa).

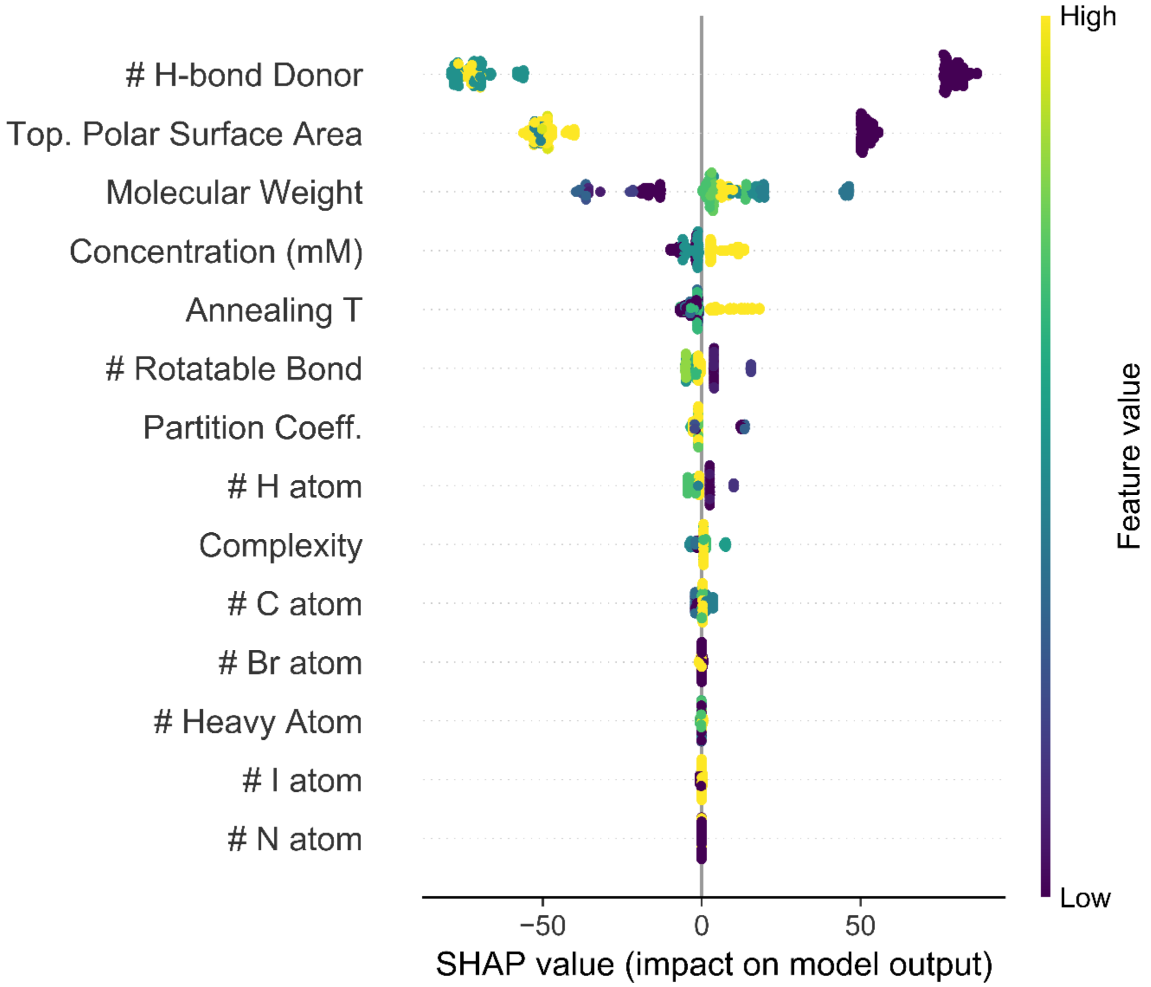

Supplementary Figure 17. The feature importance rank generated using SHAP and gradient boosting with decision trees (GB, non-normalized) for the molecular properties determining the time-intercept/ degradation onset of the capped films. The *x*-axis corresponds to the model output (higher means improving the stability, and vice versa).

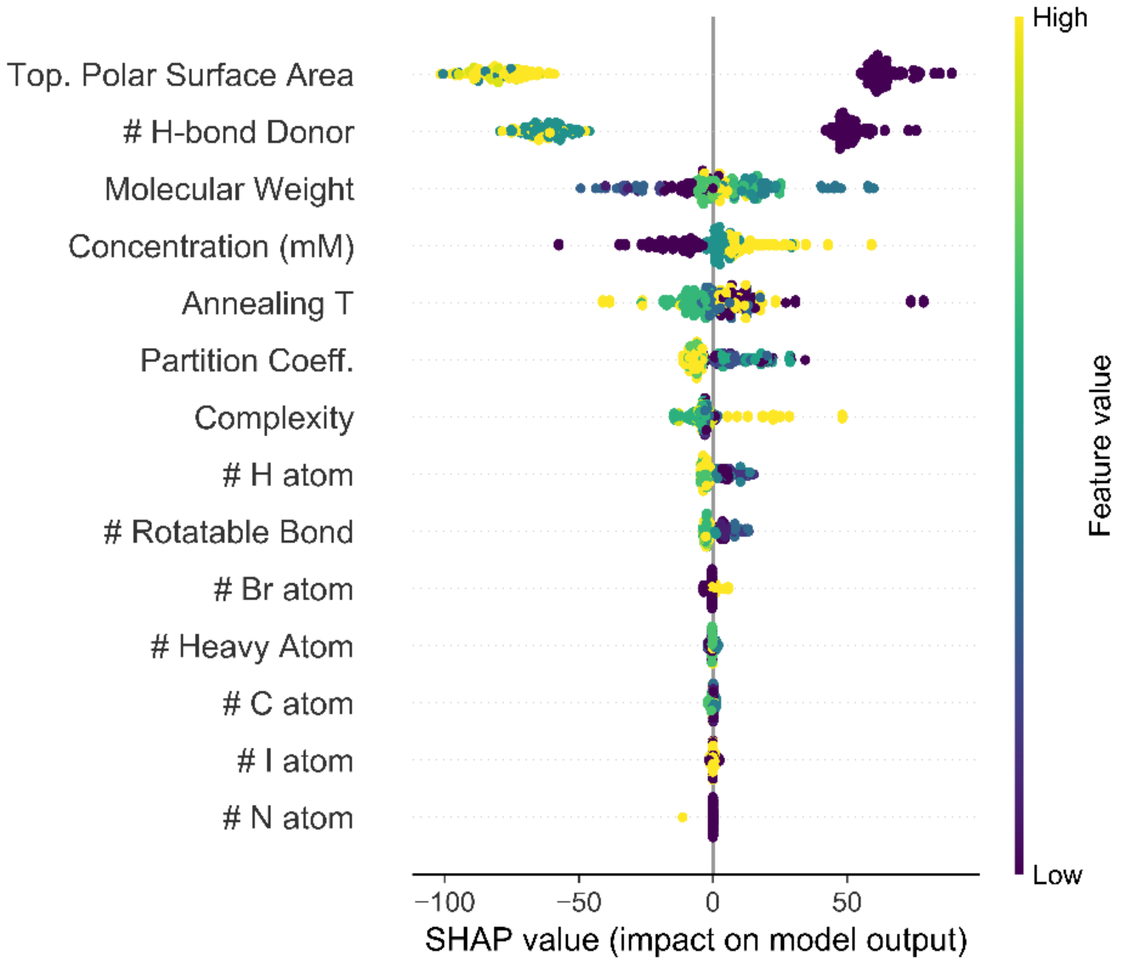

Supplementary Figure 18. The feature importance rank generated using SHAP and gradient boosting with decision trees (GB, non-normalized) for the molecular properties determining the time-intercept/ degradation onset of the capped films, and excluding the top stable capping layer material, PTEAI. The *x*-axis corresponds to the model output (higher means improving the stability, and vice versa).

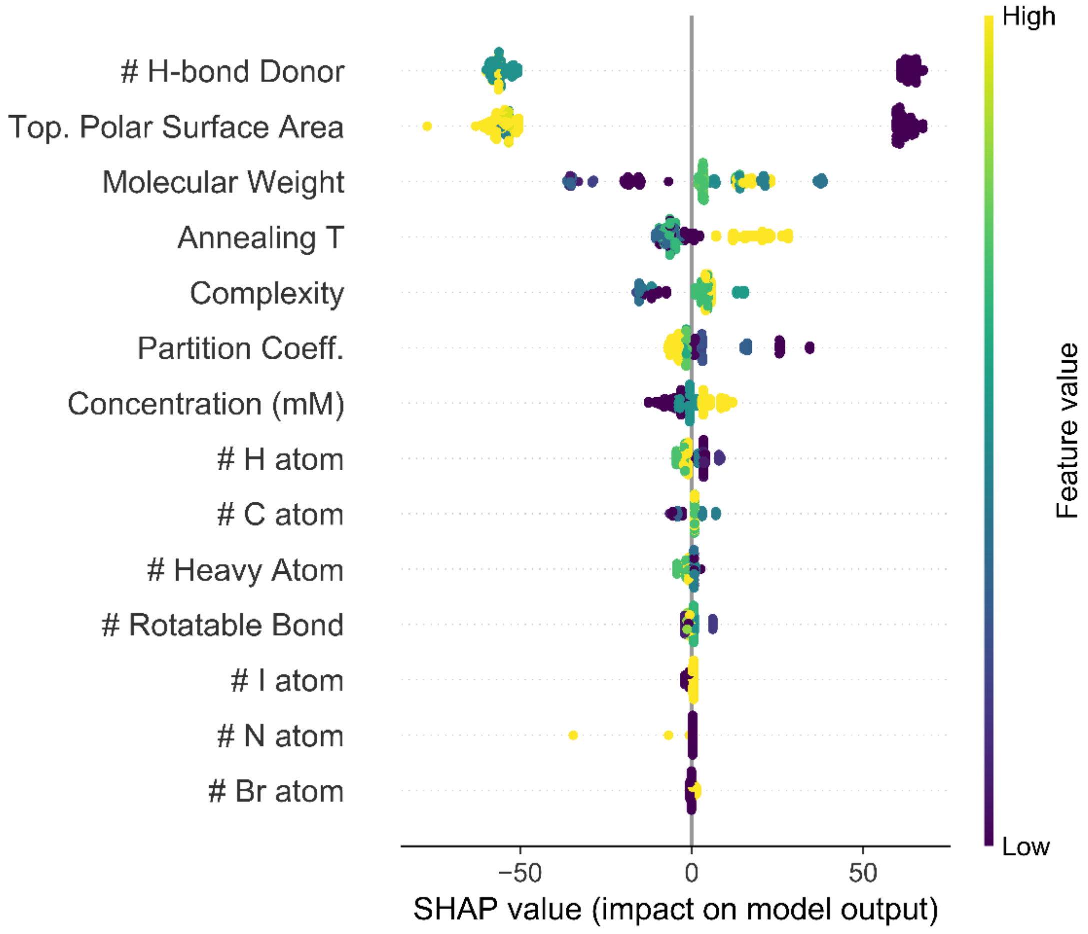

Supplementary Figure 19. Powder x-ray diffraction (XRD) spectra of pre-degraded bare and PTEAI-capped $MAPbI_3$ compared to reference $MAPbI_3$,[7] PbI_2,[8] and 2D perovskites $(BA)_2(MA)Pb_2I_7$ (n = 2), $(BA)_2(MA)_2Pb_3I_{10}$ (n = 3), and $(BA)_2(MA)_3Pb_4I_{13}$ (n = 4).[9] Based on the reference patterns, the 2D perovskites in the capping layer likely consist of $(PTEA)_2(MA)_3Pb_4I_{13}$ (n = 4), as shown in red shaded area. n indicates the number of 3D perovskite layers before separated by organic molecules, and the RP perovskite formula is in the form of $A'_2A_{n-1}B_nX_{3n+1}$. The extra peaks on bare $MAPbI_3$ comes from excess PbI_2.

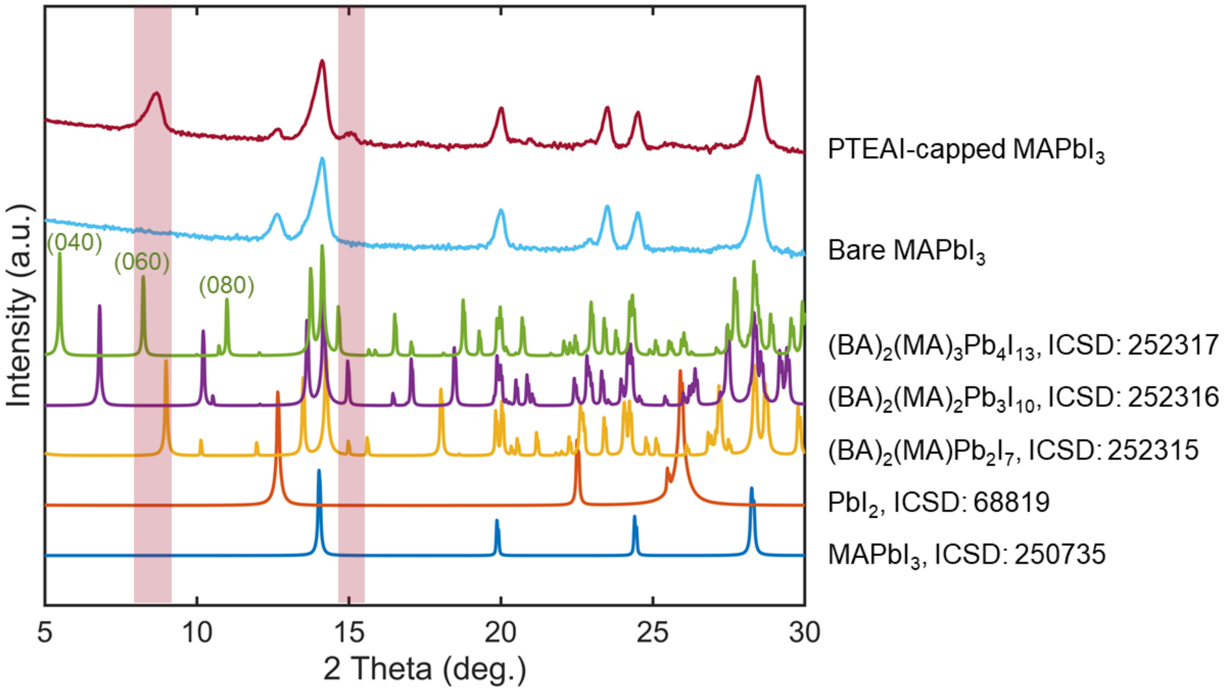

Supplementary Figure 20. Powder x-ray diffraction (XRD) data of pre-degraded bare and TPAI-capped $MAPbI_3$ compared to reference $MAPbI_3$,[7] PbI_2,[8] and 2D perovskites $(BA)_2(MA)Pb_2I_7$ (n = 2), $(BA)_2(MA)_2Pb_3I_{10}$ (n = 3), and $(BA)_2(MA)_3Pb_4I_{13}$ (n = 4).[9] Based on the reference patterns, the 2D perovskites in the capping layer consists of $(TPA)_2(MA)_2Pb_3I_{10}$ (n = 3).

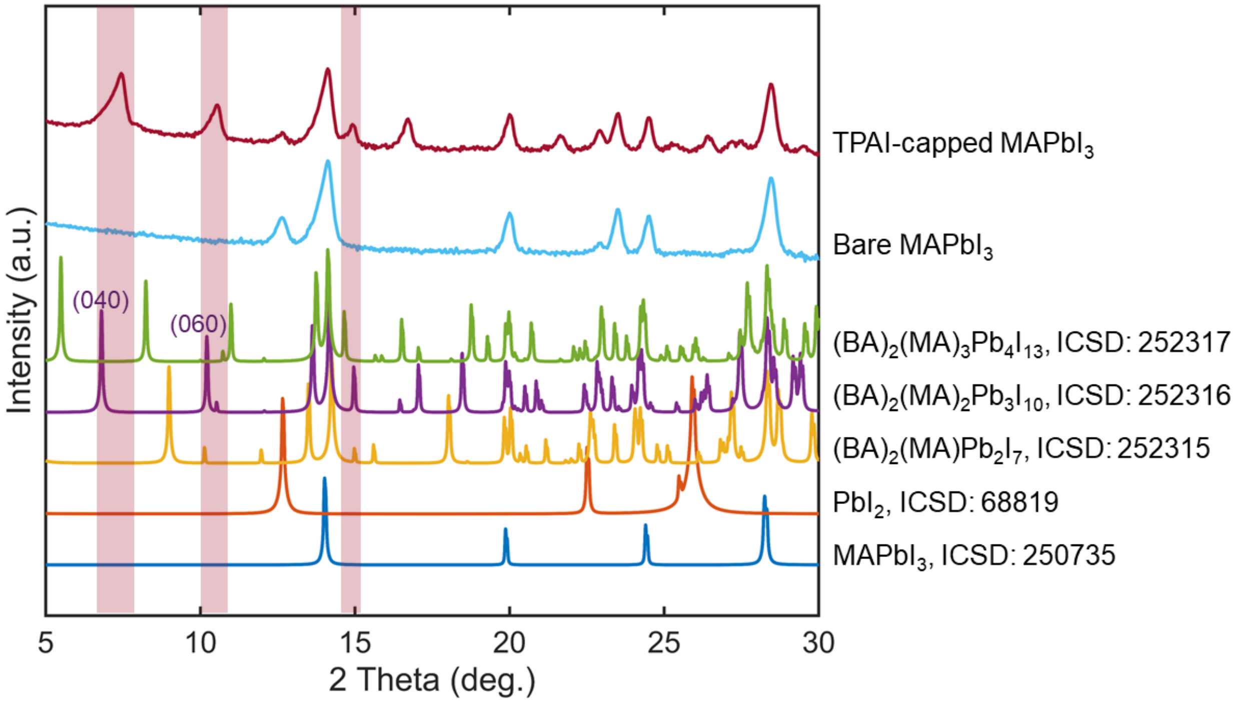

Supplementary Figure 21. Aging tests, changes of phase: Powder x-ray diffraction (XRD) data for bare $MAPbI_3$, TPAI-capped, and PTEAI-capped $MAPbI_3$. The amount of PbI_2 in the fresh samples of capping layers is reduced in comparison to fresh bare $MAPbI_3$.

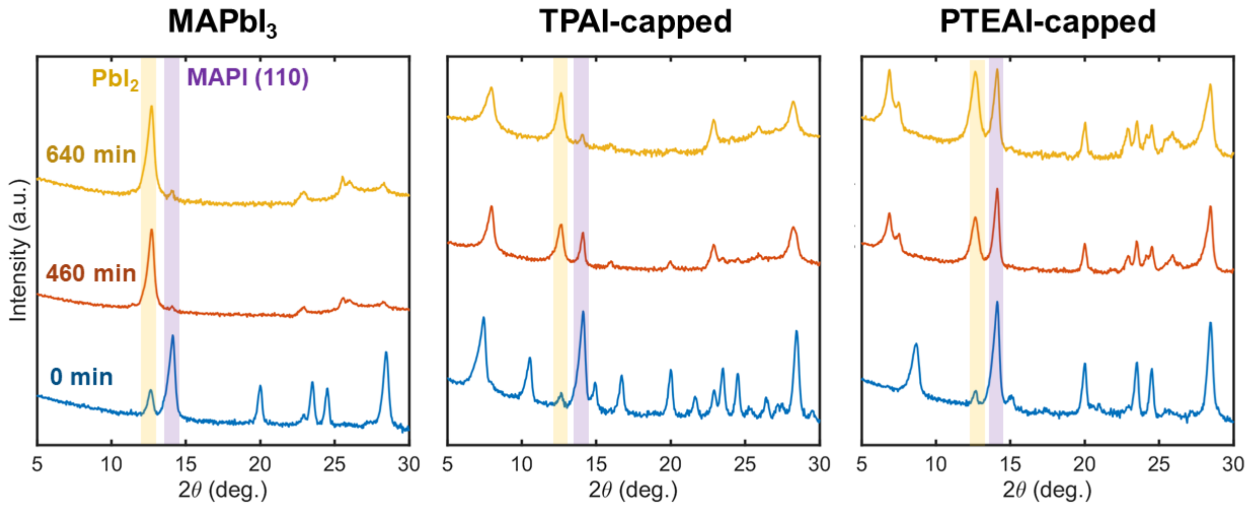

Supplementary Figure 22. Aging tests, microstructure: SEM images for bare, TPAI-capped, and PTEAI-capped $MAPbI_3$ films at 0, 460, and 640 minutes of degradation. Scale bar: 200 nm.

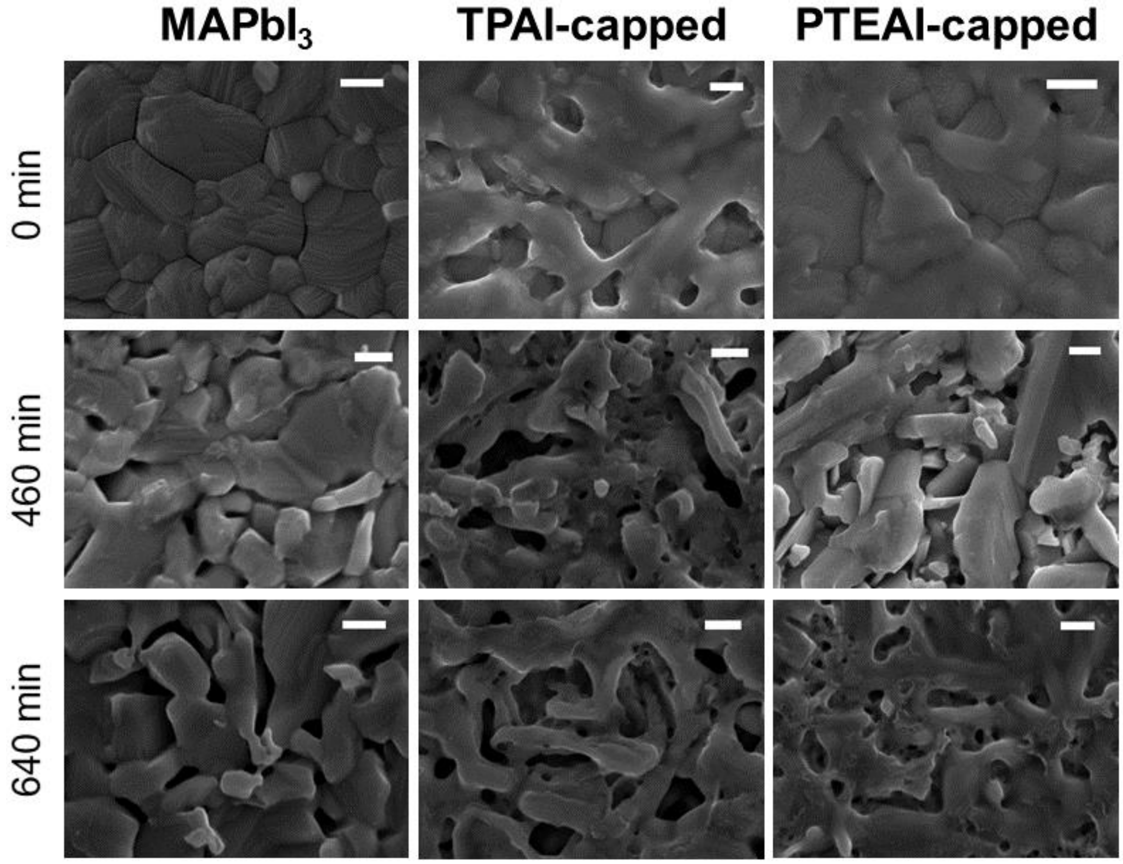

Supplementary Figure 23. Grazing incidence wide-angle x-ray scattering (GIWAXS) images for bare $MAPbI_3$, TPAI-, and PTEAI-capped films, with two incident angles of 0.12° and 0.2°, showing the structure on the surface and in the bulk films, respectively.

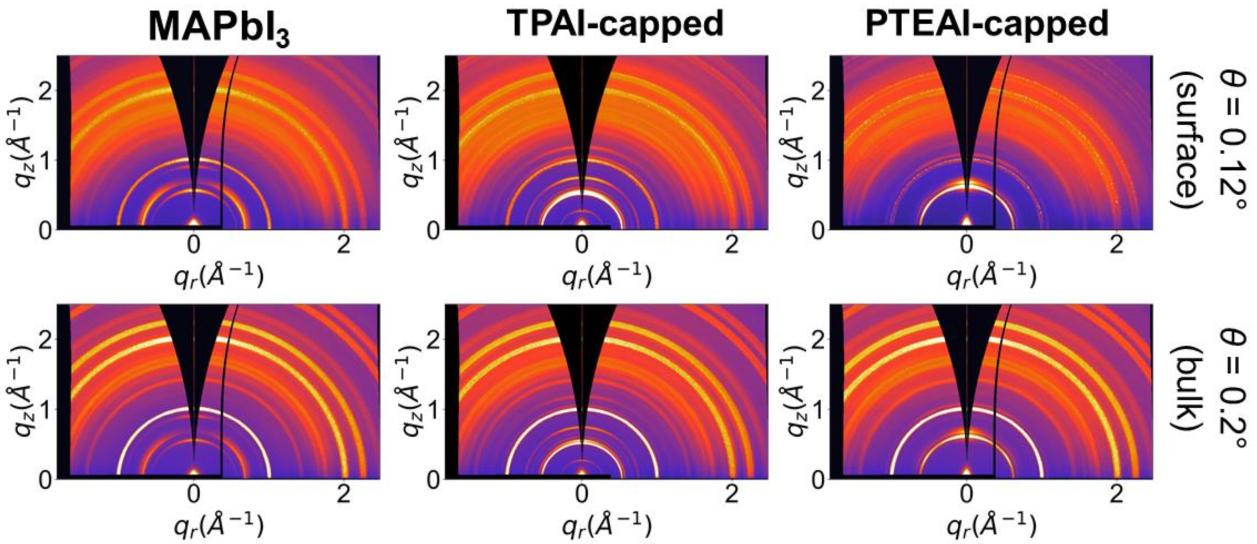

Supplementary Figure 24. Integrated plots along q_z (vertical) and q_r (horizontal) of GIWAXS data for pre-degraded bare, TPAI-capped, and PTEAI-capped $MAPbI_3$ films, normalized based on perovskite peak at $q \approx 0.98$ Å^{-1}, and their comparison with the butylammonium (BA)-based reference patterns.

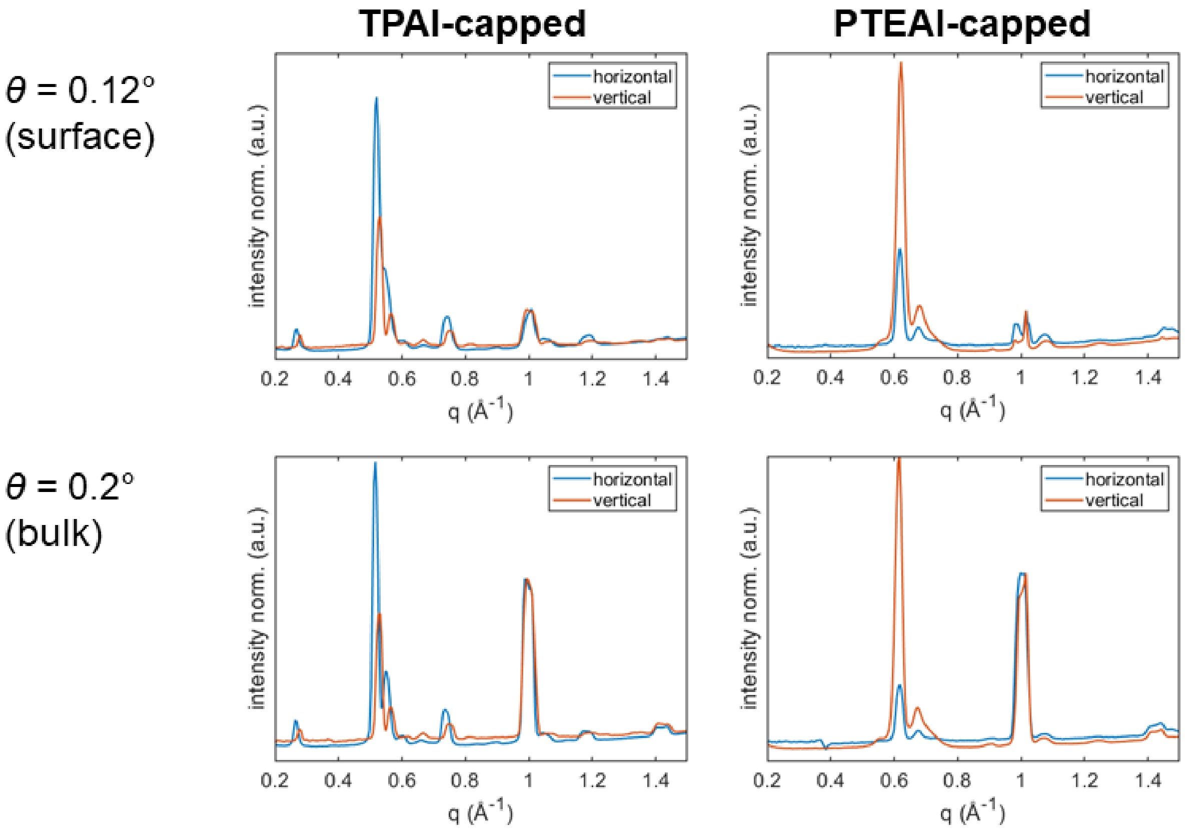

Supplementary Figure 25. The comparison between horizontal and vertical integration of TPAI-capped sample GIWAXS data at θ = 0.12° with the reference peaks. The red shade shows the LD perovskite, and the blue shade shows the TPAI precursor powder peaks.

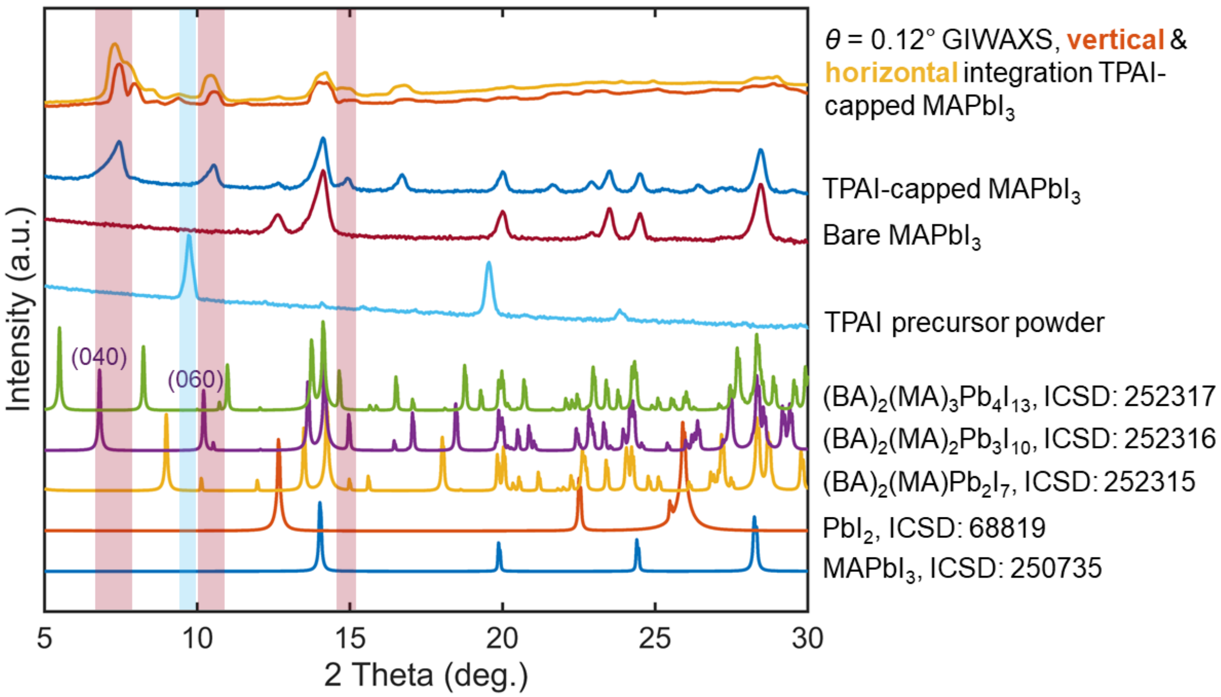

Supplementary Figure 26. The comparison between horizontal and vertical integration of PTEAI-capped sample GIWAXS data at $\theta = 0.12°$ with the reference peaks. The red shade shows the LD perovskite, and the blue shade shows the PTAI precursor powder peaks. The GIWAXS data shows the presence of precursor.

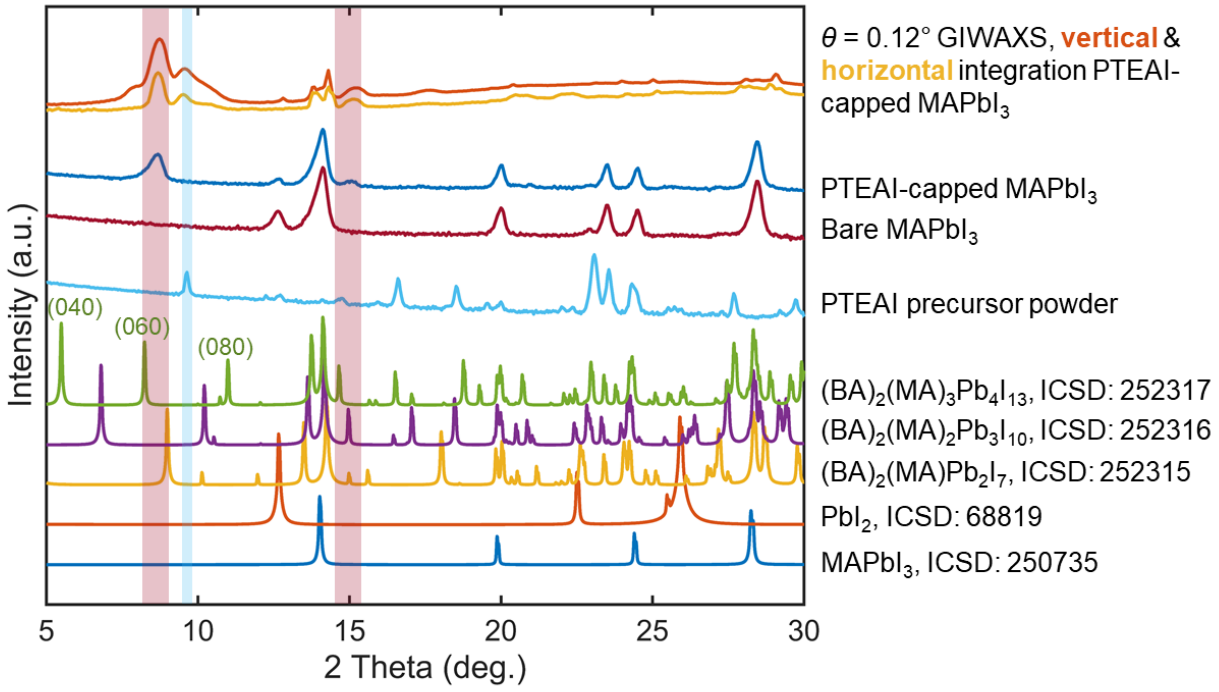

Supplementary Figure 27. X-ray photoelectron spectroscopy (XPS) data for bare, TPAI-capped, and PTEAI-capped $MAPbI_3$. TPAI, which turned yellow and its $MAPbI_3$ phase converted into PbI_2, has only one doublet left at the same binding energy as degraded $MAPbI_3$, 138.2 eV.

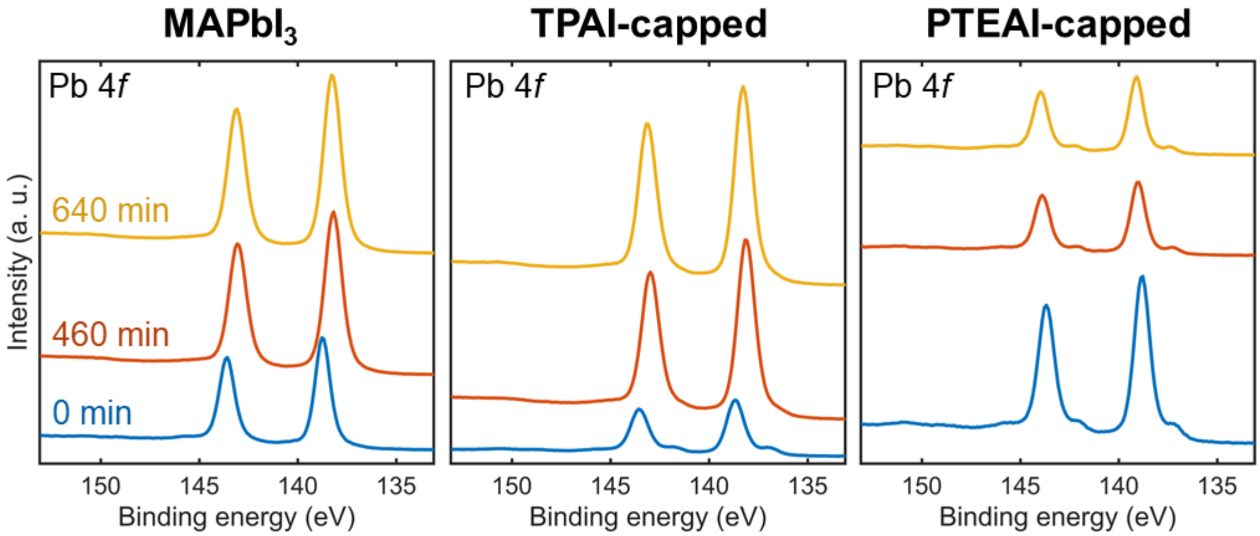

Supplementary Figure 28. The average difference of respective RGB color values between .jpg and .bmp files of the 28 random samples' aging test images, showing negligible difference (< 1).

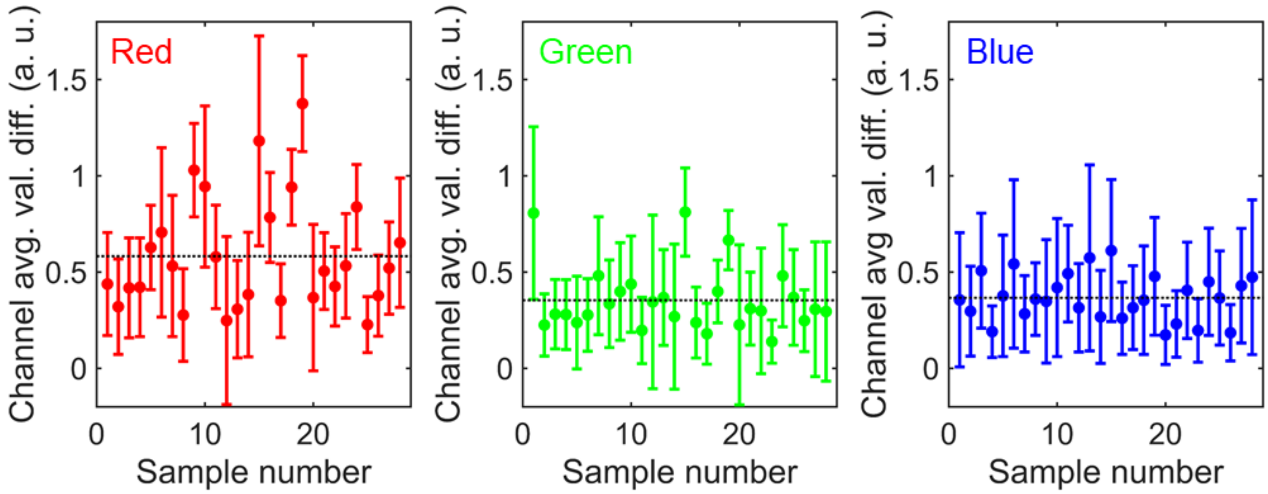

Supplementary Figure 29. The Fourier-transform infrared spectroscopy results in attenuated total reflection (ATR) geometry with ZnSe crystal for bare $MAPbI_3$, TPAI-, and PTEAI-capped films. In TPAI- and PTEAI-capped, the N^+–C stretch appears at around 3020 cm^{-1}.[10]

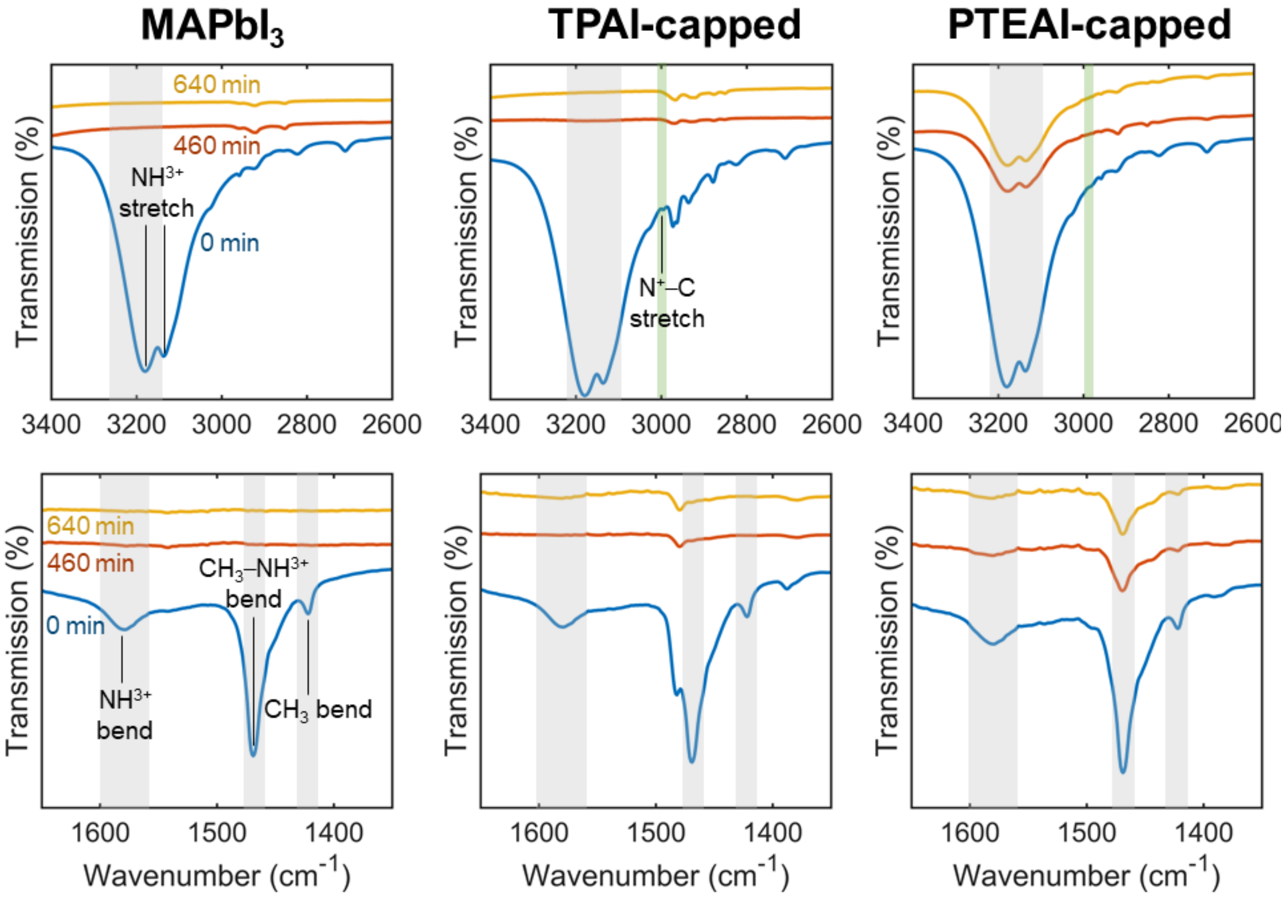

Supplementary Figure 30. The Fourier-transform infrared spectroscopy results in attenuated total reflection (ATR) geometry with Ge crystal for bare $MAPbI_3$, TPAI-, and PTEAI-capped films.

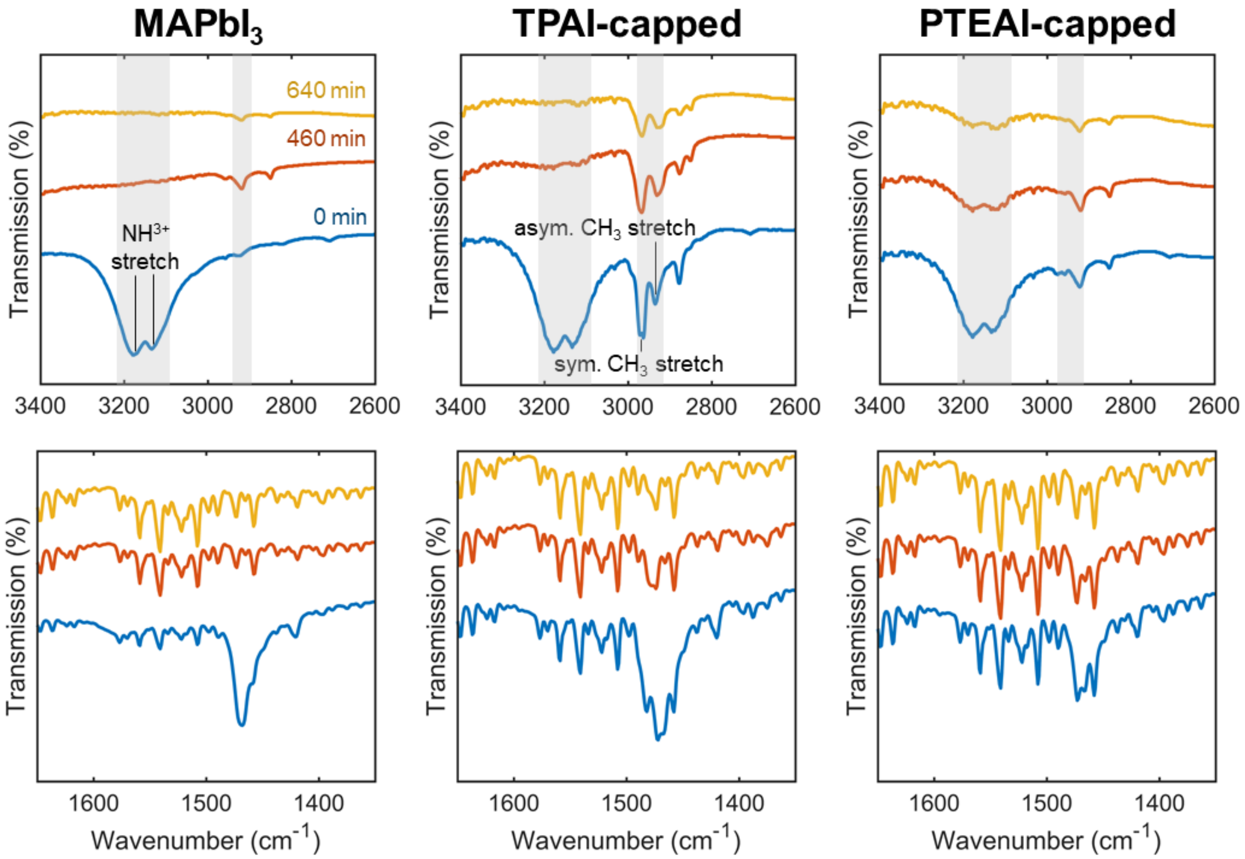

Supplementary Figure 31. Fourier-transform infrared spectroscopy (FTIR) in attenuated total reflection (ATR) geometry with zinc selenide (ZnSe) crystal for bare and PTEAI-capped $MAPbI_3$. Additional dips in the PTEAI-capped $MAPbI_3$ show the C=C stretch modes associated with the phenyl (benzene ring) functional group.

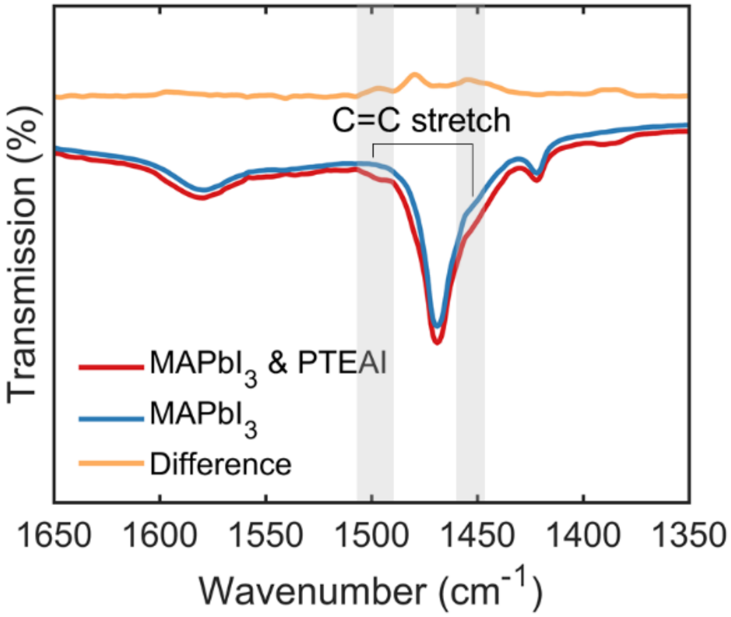

Supplementary Figure 32. The red onsets of 4 types of *A*-site cations: DA, PTEA, TBA, and TPA, with Br and I, at different processing conditions. $MAPbI_3$ has large variance, and as the onset increases, the slope decreases / the change in color is slower.

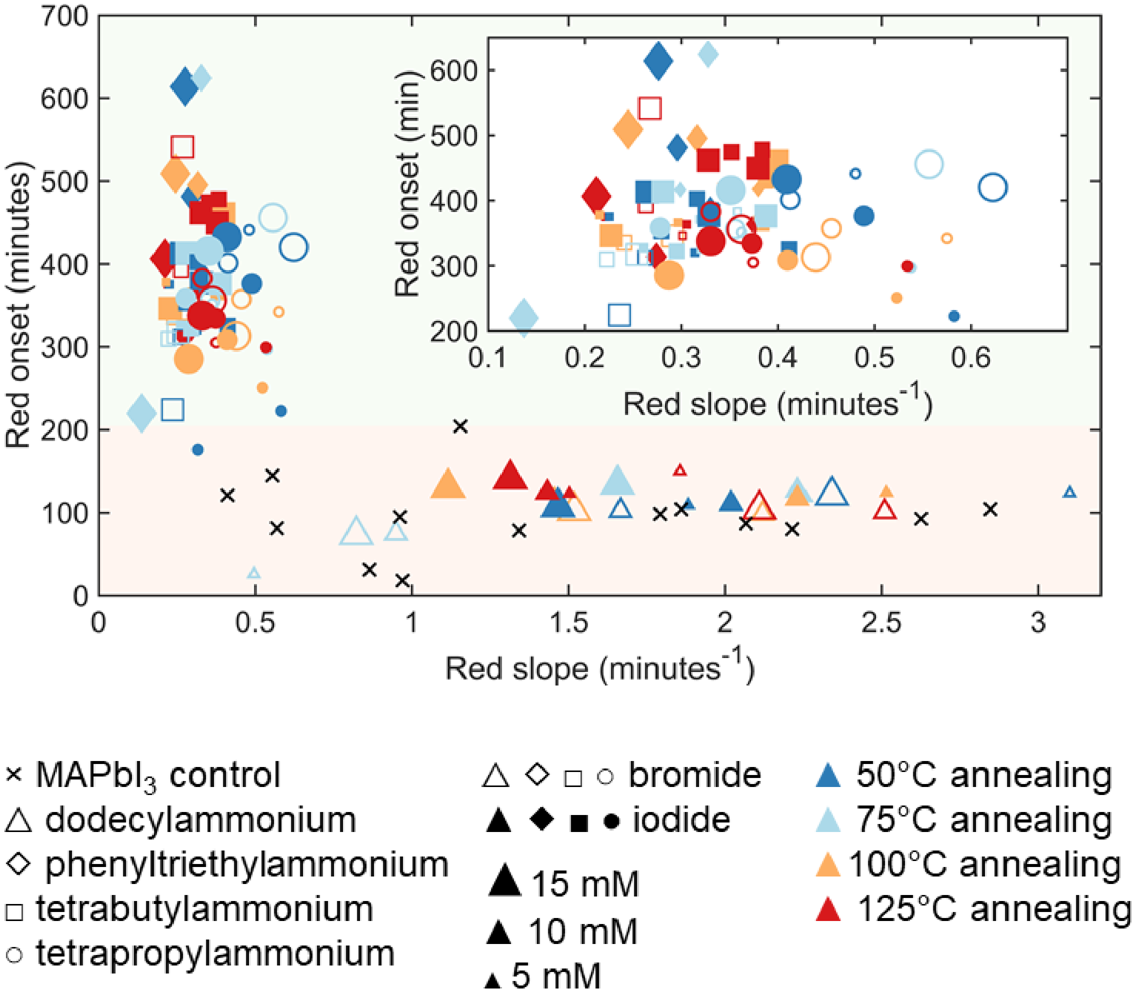

Supplementary Figure 33. Integrated circular average of GIWAXS images for bare, TPAI-capped, and PTEAI-capped $MAPbI_3$ films at different aging test time points. The shaded purple area indicates the $MAPbI_3$ peak.

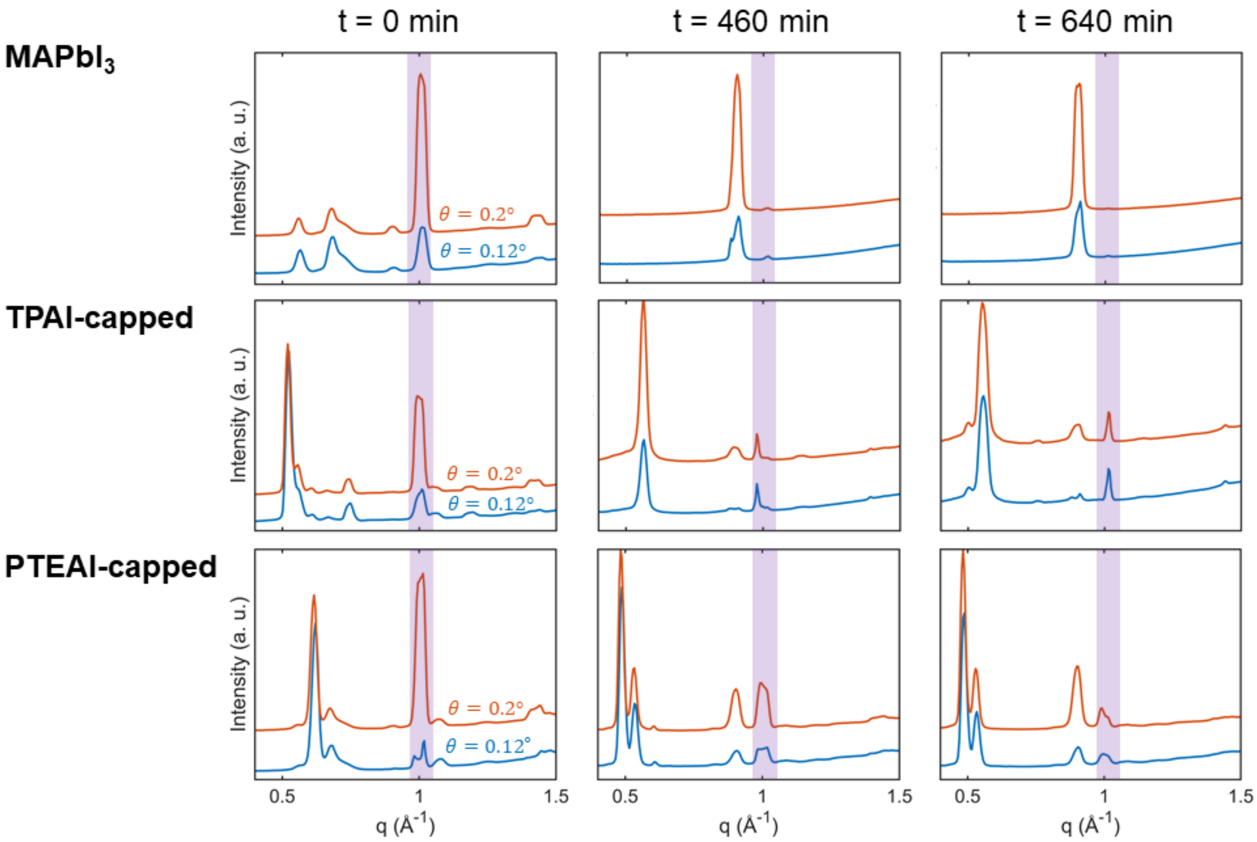

Supplementary Figure 34. The time-intercept R data/ red onset/ xinterp_r (**a**) and the slope R data/ param1_r (**b**) for the various capping layer materials. Each capping material's boxplot represents the data points across all the processing conditions with various precursor solution concentrations and annealing temperatures.

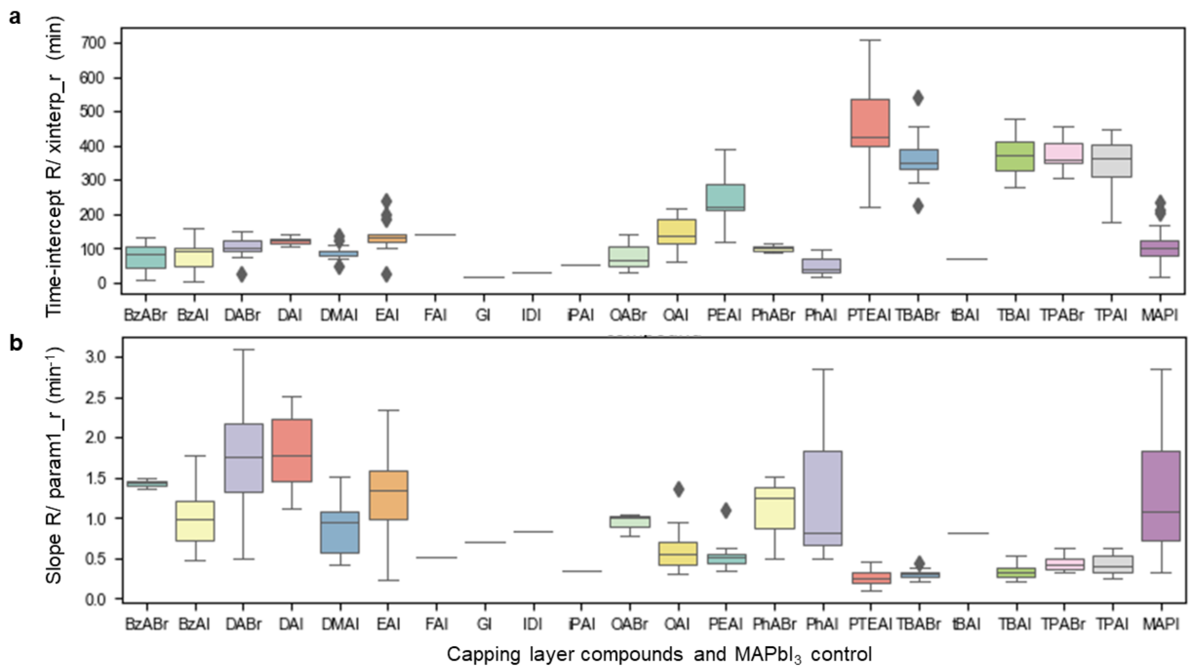

Supplementary Figure 35. The correlation between the time-intercept R the slope of R, with the Pearson correlation value of -0.61.

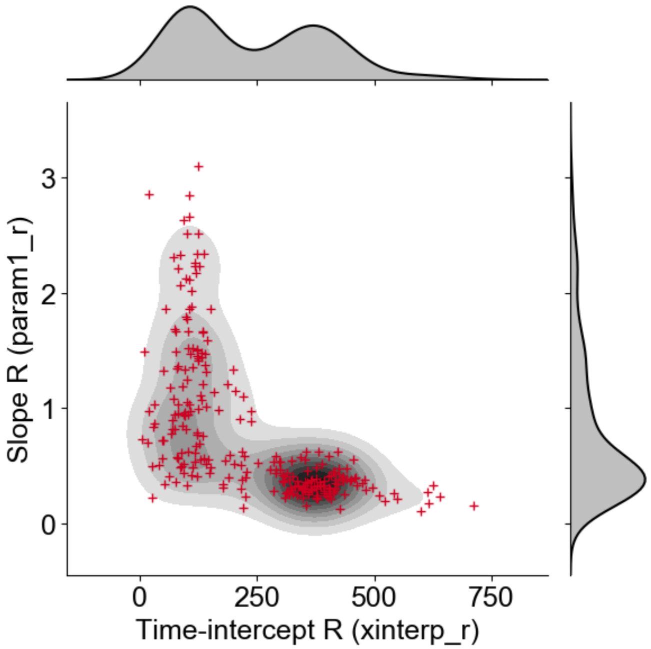

Supplementary Figure 36. The time-intercept R data/ xinterp_r for the trained data, excluding PTEAI (**a**) and the prediction based on the trained random forest regressor (**b**). Each capping material's boxplot represents the data points across all the processing conditions with various precursor solution concentrations and annealing temperatures.

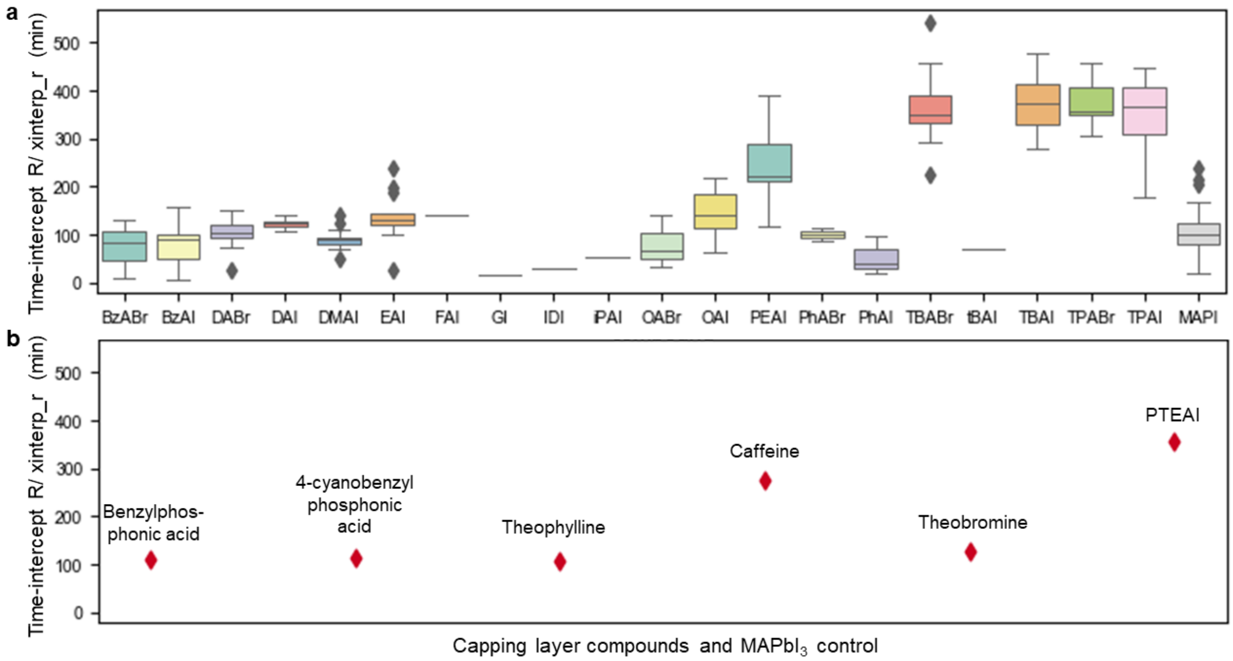

Supplementary Figure 37. A typical red, green, blue (RGB) curve from bare $MAPbI_3$ and PTEAI-capped films.

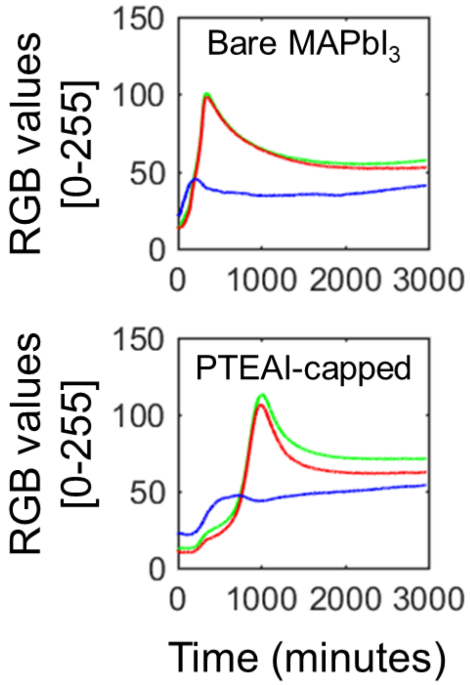

Supplementary Tables

Supplementary Table 1. List of capping layer materials explored in this study, and their PubChem ID's.[11]

#	Compound name	Molecular formula	Cation PubChem CID	Compound PubChem CID
1	Benzylammonium bromide	$C_7H_{10}NBr$	4996382	12998568
2	Benzylammonium iodide	$C_7H_{10}NI$	4996382	21983538
3	Dimethylammonium iodide	C_2H_8NI	3614769	12199010
4	Ethylammonium iodide	C_2H_8NI	3427831	11116533
5	Formamidinium iodide	CH_5N_2I	59979088	89906651
6	Guanidinium iodide	CH_6N_3I	32838	129654296
7	Imidazolium iodide	$C_3H_5N_2I$	444234	12203051
8	*Iso*-propylammonium iodide	$C_3H_{10}NI$	3364502	91972165
9	*n*-dodecylammonium bromide	$C_{12}H_{28}NBr$	3390094	21872287
10	*n*-dodecylammonium iodide	$C_{12}H_{28}NI$	3390094	88495928
11	*n*-octylammonium bromide	$C_8H_{20}NBr$	3691730	22247282
12	*n*-octylammonium iodide	$C_8H_{20}NI$	3691730	22461615
13	Phenylammonium bromide	C_6H_8NBr	3713298	68330
14	Phenylammonium iodide	C_6H_8NI	3713298	6450296
15	Phenylethylammonium iodide	$C_8H_{12}NI$	448751	23297368
16	Phenyltriethylammonium iodide	$C_{12}H_{20}NI$	70525	70524
17	*Tert*-butylammonium iodide	$C_4H_{12}NI$	3833866	22344722
18	Tetrabutylammonium bromide	$C_{16}H_{36}NBr$	16028	74236
19	Tetrabutylammonium iodide	$C_{16}H_{36}NI$	16028	67553
20	Tetrapropylammonium bromide	$C_{12}H_{28}NBr$	9559	74745
21	Tetrapropylammonium iodide	$C_{12}H_{28}NI$	9559	12429

Supplementary Table 2. The machine learning algorithms and their RMSE's for *normalized* input (*X*).

Regression algorithms	Hyperparameters in scikit-learn	Train RMSE (minutes)	Test RMSE (minutes)	Cross-validated RMSE (minutes)
Linear		61.8	84.0	3.04E13
K-nearest neighbor	{'algorithm': 'ball_tree', 'n_neighbors': 3, 'weights': 'uniform'}	48.4	90.3	116.7
Random forest	{'max_depth': 4, 'n_estimators': 30}	46.4	70.8	105.4
Gradient boosting	{'max_depth': 4, 'n_estimators': 40}	39.8	81.9	115.7
Neural network (multilayer perceptron)	{hidden_layer_sizes = (128,256,64,), max_iter = 1000, learning_rate = 'constant', solver = 'adam', alpha = 0.01, activation='relu'}	64.3	84.2	161.2
Support vector machine	{'C': 500.0}	41.3	142	97.3

Supplementary Table 3. The machine learning algorithms, their optimized hyperparameters, and their cross-validated RMSE's for *non-normalized* input (*X*).

**Regression algorithms**	**Hyperparameters in scikit-learn**	**Train RMSE (minutes)**	**Test RMSE (minutes)**	**Cross-validated RMSE (minutes)**
Linear		61.8	84.0	164.3
K-nearest neighbor	{'algorithm': 'ball_tree', 'n_neighbors': 3, 'weights': 'uniform'}	46.4	91.2	148.3
Random forest	{'max_depth': 4, 'n_estimators': 60}	46.5	70.8	104.8
Gradient boosting	{'max_depth': 4, 'n_estimators': 30}	41.7	78.5	112.3
Neural network (multilayer perceptron)	{hidden_layer_sizes =(128,256,64),max_iter=10000, learning_rate='constant', solver='adam', alpha=0.1, activation='relu'}	44.5	89.2	132.4
Support vector machine	{'C': 1000.0}	41.3	142	169.0

Supplementary Table 4. The statistics of the film samples for the red (R) slope and red (R) time-intercept/ degradation onset. Note that for FAI, GI, IDI, iPAI, tBAI, there is only one film each, fabricated using 10 mM precursor solution concentration and annealed at 100°C.

Compound	Slope R Mean (minutes)	Slope R Std. Dev. (minutes)	Time-intercept R Mean (minutes)	Time-intercept R Std. Dev. (minutes)	Number of films
BzABr	1.43	0.05	74	51	3
BzAI	1.00	0.35	81	39	12
DABr	1.75	0.72	100	30	12
DAI	1.80	0.43	122	10	12
DMAI	0.90	0.35	91	23	13
FAI					1
GI					1
IDI					1
iPAI					1
tBAI					1
OABr	0.95	0.11	80	46	3
OAI	0.62	0.27	145	45	15
PhABr	1.09	0.43	100	10	3
PhAI	1.39	1.04	52	33	3
TBABr	0.31	0.05	363	60	24
TBAI	0.33	0.08	376	55	28
TPABr	0.44	0.09	374	46	12
TPAI	0.43	0.12	353	66	28
EAI	1.34	0.53	136	49	13
PEAI	0.54	0.19	241	68	12
PTEAI	0.27	0.10	462	115	27
Bare $MAPbI_3$	1.29	0.71	107	45	35
**Total film samples**					**260**

Supplementary Table 5. List of capping layer material manufacturers used in this study.

#	Compound name	Molecular formula	Manufacturer
1	Benzylammonium bromide	$C_7H_{10}NBr$	Sigma-Aldrich
2	Benzylammonium iodide	$C_7H_{10}NI$	Sigma-Aldrich
3	Dimethylammonium iodide	C_2H_8NI	Greatcell Solar
4	Ethylammonium iodide	C_2H_8NI	Greatcell Solar
5	Formamidinium iodide	CH_5N_2I	Greatcell Solar
6	Guanidinium iodide	CH_6N_3I	Greatcell Solar
7	Imidazolium iodide	$C_3H_5N_2I$	Greatcell Solar
8	*Iso*-propylammonium iodide	$C_3H_{10}NI$	Greatcell Solar
9	*n*-dodecylammonium bromide	$C_{12}H_{28}NBr$	TCI America
10	*n*-dodecylammonium iodide	$C_{12}H_{28}NI$	TCI America
11	*n*-octylammonium bromide	$C_8H_{20}NBr$	Lumtec
12	*n*-octylammonium iodide	$C_8H_{20}NI$	Lumtec
13	Phenylammonium bromide	C_6H_8NBr	Sigma-Aldrich
14	Phenylammonium iodide	C_6H_8NI	Sigma-Aldrich
15	Phenylethylammonium iodide	$C_8H_{12}NI$	TCI America
16	Phenyltriethylammonium iodide	$C_{12}H_{20}NI$	Sigma-Aldrich
17	*Tert*-butylammonium iodide	$C_4H_{12}NI$	Greatcell Solar
18	Tetrabutylammonium bromide	$C_{16}H_{36}NBr$	Sigma-Aldrich
19	Tetrabutylammonium iodide	$C_{16}H_{36}NI$	Sigma-Aldrich
20	Tetrapropylammonium bromide	$C_{12}H_{28}NBr$	Sigma-Aldrich
21	Tetrapropylammonium iodide	$C_{12}H_{28}NI$	Sigma-Aldrich

Supplementary Table 6. The top 5 SHAP features for linear, random forest, and gradient boosting decision tree regressions, with the complete dataset and PTEAI-capped-excluded dataset.

**Machine learning models**	**Top 5 SHAP features, dataset: all**	**Top 5 SHAP features, dataset: excluding PTEAI-capped**
Random forest regression	1. Top. polar surface area<br>2. # H-bond donor<br>3. Molecular weight<br>4. Concentration<br>5. Partition coefficient	1. # H-bond donor<br>2. Top. polar surface area<br>3. Molecular weight<br>4. Concentration<br>5. Annealing temperature
Gradient boosting regression with decision trees	1. Top. polar surface area<br>2. # H-bond donor<br>3. Molecular weight<br>4. Concentration<br>5. Annealing temperature	1. # H-bond donor<br>2. Top. polar surface area<br>3. Molecular weight<br>4. Annealing temperature<br>5. Complexity
Linear regression (non-normalized)	1. Molecular weight<br>2. # Heavy atom<br>3. # H atom<br>4. # Complexity<br>5. # I atom	1. Molecular weight<br>2. # H atom<br>3. # Heavy atom<br>4. Complexity<br>5. # Rotatable bond

Supplementary References

1. Pedregosa, F. *et al.* Scikit-learn: Machine Learning in Python. (2012). http://arxiv.org/abs/1201.0490.
2. Aristidou, N. *et al.* Fast oxygen diffusion and iodide defects mediate oxygen-induced degradation of perovskite solar cells. *Nat. Commun.* **8**, 1–10 (2017).
3. Fan, Z. *et al.* Layer-by-Layer Degradation of Methylammonium Lead Tri-iodide Perovskite Microplates. *Joule* **1**, 548–562 (2017).
4. Gao, P., Bin Mohd Yusoff, A. R. & Nazeeruddin, M. K. Dimensionality engineering of hybrid halide perovskite light absorbers. *Nat. Commun.* **9**, 5028 (2018).
5. Gao, L. *et al.* Improved Environmental Stability and Solar Cell Efficiency of (MA,FA)PbI $_3$ Perovskite Using a Wide-Band-Gap 1D Thiazolium Lead Iodide Capping Layer Strategy. *ACS Energy Lett.* **4**, 1763–1769 (2019).
6. Menesatti, P. *et al.* RGB Color Calibration for Quantitative Image Analysis: The "3D Thin-Plate Spline" Warping Approach. *Sensors* **12**, 7063–7079 (2012).
7. Stoumpos, C. C., Malliakas, C. D. & Kanatzidis, M. G. Semiconducting Tin and Lead Iodide Perovskites with Organic Cations: Phase Transitions, High Mobilities, and Near-Infrared Photoluminescent Properties. *Inorg. Chem.* **52**, 9019–9038 (2013).
8. Palosz, B. The structure of PbI2polytypes 2H and 4H: A study of the 2H-4H transition. *J. Phys. Condens. Matter* **2**, 5285–5295 (1990).
9. Stoumpos, C. C. *et al.* Ruddlesden–Popper Hybrid Lead Iodide Perovskite 2D Homologous Semiconductors. *Chem. Mater.* **28**, 2852–2867 (2016).
10. Anastassopoulou, J. D. Mass and FT-IR Spectra of Quaternary Ammonium Surfactants. in 1–9 (1991). doi:10.1007/978-94-011-3620-4_1
11. Kim, S. *et al.* PubChem 2019 update: improved access to chemical data. *Nucleic Acids Res.* **47**, D1102–D1109 (2019).
