# Supplementary material for: How machine learning can help select capping layers to suppress perovskite degradation
Source: Nat Commun. 2020 Aug 20;11:4172. doi: 10.1038/s41467-020-17945-4 (PMC7441172; doi:10.1038/s41467-020-17945-4)
Supplement: Supplementary file 3 — Description of Additional Supplementary Files [file 41467_2020_17945_MOESM3_ESM.pdf]

Description of Additional Supplementary Files

File name: Supplementary Data 1

Description: Raw data of RGB colour measurements and extracted degradation onsets.
